# Synthetic Periodontal Guided Tissue Regeneration Membrane with Self‐Assembling Biphasic Structure and Temperature‐Sensitive Shape Maintenance

**DOI:** 10.1002/adhm.202402137

**Published:** 2024-10-23

**Authors:** W. Benton Swanson, Seth M. Woodbury, Renan Dal‐Fabbro, Lindsey Douglas, Jackson Albright, Miranda Eberle, David Niemann, Jinping Xu, Marco C. Bottino, Yuji Mishina

**Affiliations:** ^1^ Department of Biologic and Materials Science School of Dentistry University of Michigan Ann Arbor MI 48109 USA; ^2^ Department of Chemistry College of Literature Science and the Arts University of Michigan Ann Arbor MI 48109 USA; ^3^ Department of Physics College of Literature Science and the Arts University of Michigan Ann Arbor MI 48109 USA; ^4^ Department of Cariology Restorative Sciences and Endodontics School of Dentistry University of Michigan Ann Arbor MI 48109 USA; ^5^ Department of Biomedical Engineering College of Engineering University of Michigan Ann Arbor MI 48109 USA; ^6^ Present address: Department of Oral Medicine Infection and Immunology Harvard School of Dental Medicine Boston MA 02115 USA

**Keywords:** bone, membrane, network polymer, periodontal ligament, periodontal regeneration, tissue engineering

## Abstract

Periodontal disease poses significant challenges to the long‐term stability of oral health by destroying the supporting structures of teeth. Guided tissue regeneration techniques, particularly barrier membranes, enable local regeneration by providing an isolated, protected compartment for osseous wound healing while excluding epithelial tissue. Here, this study reports on a thermosensitive periodontal membrane (TSPM) technology designed to overcome the mechanical limitations of current membranes through a semi‐interpenetrating network of high molecular weight poly(L‐lactic acid) (PLLA) and in situ‐polymerized mesh of poly(ε‐caprolactone)diacrylate (PCL‐DA), and poly lactide‐co‐glycolide diacrylate (PLGA‐DA). An optimized composition allows facile reshaping at greater than 52 °C and rigid shape maintenance at physiological temperature. Its unique bilayer morphology is achieved through self‐assembly and thermally‐induced phase separation, resulting in distinct yet continuous smooth and nanofibrous compartments adequate for epithelial occlusion and regeneration. Incorporating PLGA‐DA enhances the membrane's hydrophilicity and degradation properties, facilitating a more rapid and controlled degradation and therapeutic delivery. This study demonstrates its ability to promote local regeneration by serving as a barrier membrane and simultaneously as a scaffolding matrix in a rat orthotopic periodontal defect model. The TSPM outperformed a clinically available material (Epi‐Guide) to facilitate robust alveolar bone and periodontal ligament regeneration at 4 and 8 weeks.

## Graphical abstract

Periodontal disease challenges oral health by damaging tooth support structures. Newly developed thermosensitive periodontal membranes (TSPM) utilize a blend of poly(L‐lactic acid) and polymerized poly(ε‐caprolactone)diacrylate in a semi‐interpenetrating mesh, enabling improved handling properties and facilitating effective wound healing. These membranes promote alveolar bone and periodontal ligament regeneration, outperforming existing materials in a rat model at 4 and 8 weeks.

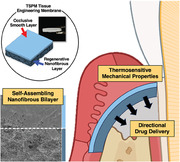

## 1 Introduction

Periodontal disease, a prevalent condition affecting the supporting structures of the teeth, leads to the destruction of the periodontal ligament, alveolar bone, and cementum. The disease's progression can result in tooth mobility and eventual loss, significantly impacting oral function and overall health.^[^ ^1^ ^]^ The management of periodontal disease often involves surgical regenerative procedures to restore the lost periodontal tissues to their original architecture and function. Guided tissue regeneration (GTR) has emerged as a pivotal technique in periodontal therapy, utilizing barrier membranes to direct the growth of new bone and periodontal ligament by excluding fast‐migrating epithelial and connective tissue cells from the healing area.^[^ ^2^, ^3^, ^4^, ^5^, ^6^, ^7^ ^]^ The original hypothesis proposed by Dahlin and Linde was that a barrier separating the periodontal wound's slow‐growing cells from the overlying epithelium and connective tissue would promote bone formation by the slower‐growing osteoprogenitor cells.^[^ ^8^ ^]^ Various biomaterial membranes have been developed for clinical use since this initial concept of a passive barrier membrane. Recent evidence suggests that membranes may play an active role in hosting and modulating the activities of membrane‐associated cells during regeneration.^[^ ^9^ ^]^

Clinically, various membranes have been employed for GTR, ranging from non‐resorbable to resorbable types.^[^ ^4^ ^]^ Non‐resorbable barrier membranes, such as expanded polytetrafluoroethylene (ePTFE), have been widely used due to their favorable tissue response and clinical manageability.^[^ ^10^, ^11^ ^]^ However, they require a second surgical procedure for removal, which can increase surgical site morbidity.^[^ ^12^, ^13^ ^]^ Resorbable membranes, made from collagen,^[^ ^14^ ^]^ polylactic acid, and polyglycolic acid, have gained popularity as they do not require membrane removal and show good biocompatibility,^[^ ^15^, ^16^ ^]^ particularly bilayer membranes. The purpose of the bilayer, often achieved through electrospinning two meshes, is to create an outward‐facing layer toward the gingiva to prevent epithelial and connective tissue from infilling into the defect.

In contrast, the internal osteogenic layer promotes bone formation along its fibrous surface. One of the significant limitations is the inadequate mechanical properties, particularly of biodegradable ones like collagen membranes.^[^ ^14^ ^]^ These membranes generally do not possess sufficient rigidity to withstand the pressures of the surrounding tissue, leading to a collapse into the defect area. This collapse can compromise the space maintenance required for an effective bone augmentation at dimensions adequate for future dental implant placement ^[^ ^17^, ^18^ ^]^ and fall short, particularly in maintaining the horizontal and vertical dimensions of the periodontal defect.^[^ ^19^, ^20^ ^]^ Despite these advancements, the quest for an ideal membrane that combines biocompatibility, cell occlusivity, space maintenance, and clinical manageability continues. To address this limitation of current membrane technology, clinicians have employed titanium tenting screws and titanium‐reinforced membranes to provide structural support to the membrane and prevent collapse into the defect.^[^ ^21^, ^22^ ^]^ However, these methods have disadvantages.^[^ ^17^, ^22^, ^23^, ^24^, ^25^, ^26^ ^]^ Titanium tenting screws can lead to increased clinical complexity and potential complications such as screw loosening or exposure and subsequent risk of infection. While providing rigidity, titanium‐reinforced membranes can be difficult to adapt to the defect's contours and may lead to soft tissue complications if exposed.

In more significant defects, membranes may be combined with additional grafting materials or biological growth factors to stimulate regeneration within the isolated compartment.^[^ ^27^, ^28^ ^]^ The use of collagen membranes and growth factors together is based on the principle that the physical barrier provided by the membrane can protect the defect area. At the same time, the growth factors promote chemotaxis, proliferation, differentiation, neovascularization, and synthesis of proteins and extracellular matrix using clinical products such as platelet‐derived growth factor (PDGF, sold under the tradename: Gem21S) or enamel matrix derivative (EMD, sold under the tradename: Emdogain).^[^ ^29^, ^30^ ^]^ The therapeutic potential of growth factors is highly dependent on their concentration and the timing of their delivery to the regeneration site, often clearing rapidly before significant regeneration has occurred. Therefore, their unregulated administration can compromise the effectiveness of growth factors by inadequate pharmacokinetic control.^[^ ^31^, ^32^ ^]^ The lack of effective delivery systems and the challenges in controlling the release rates of growth factors can lead to mixed regenerative outcomes.

Synthetic biomaterials such as aliphatic polyesters have been exploited for various biomedical uses owing to their favorable degradation kinetics, tailorable mechanical properties, biocompatibility, and compositional modularity.^[^ ^33^ ^]^ Their synthetic nature allows additional chemical functionality to be engineered into the polymeric structure through novel monomers or end‐group functionalization to suit various applications. The development of temperature‐sensitive biodegradable materials has garnered significant interest in biomedical applications due to their unique ability to respond to thermal stimuli to modulate their mechanical properties.^[^ ^34^ ^]^ Temperature‐sensitive hydrogel technologies such as polyethylene glycol diacrylate or gelatin methacrylate have been described for various drug delivery applications in treating periodontal disease. For example, a liquid formulation at room temperature which gels to release a drug at physiologic temperature or sites of inflammation.^[^ ^35^, ^36^, ^37^ ^]^ While hydrogels conform to the shape of a well‐contained defect and have shown promising results for local regeneration or inflammation modulation, periodontal defects often warrant regenerative procedures that require dimensional augmentation beyond their current shape to restore bone adequately. To date, a barrier membrane with bimodal mechanical properties allowing for both clinical rigidity necessary to maintain a defect and elasticity sufficient for its deformation and patient‐specific customization has yet to be reported. Previously, our developed and validated a thermosensitive memorized microstructure (TS‐MMS) nanofibrous composite from high molecular weight poly (L‐lactic acid) (PLLA) and an interpenetrating network of poly (ε‐caprolactone) (PCL), in a composition aiming to address the need for conformal fitting scaffolding matrices.^[^ ^38^ ^]^ A critical partial‐melting temperature of TS‐MMS at 52 °C enabled bulk deformation above this temperature while retaining the nanofibrous structures upon cooling to 37 °C within a 3D macroporous tissue engineering scaffold.

Here, we present a novel thermosensitive periodontal membrane (TSPM) technology that overcomes clinical barriers to shape maintenance and predictable growth factor delivery. It demonstrates its ability to facilitate local regeneration by serving as a barrier membrane and simultaneously as a scaffolding matrix. Iterating on our previous PLLA/PCL TS‐MMS composite, we engineered a PLLA/PCL composite incorporating poly‐lactide‐co‐glycolide (PLGA) to improve degradation properties and biocompatibility. We developed a self‐assembly fabrication protocol to facilitate a bilayer morphology within a single, contiguous biomaterial construct where one side occludes epithelial ingrowth. The opposing side acts as a synthetic extracellular matrix to promote cell ingrowth and regeneration, specifically in the defect compartment. We demonstrate the robust thermosensitive mechanical properties of our TSPM, which is capable of both deformation and rigid shape maintenance and is controlled by a thermosensitive chemical chaperone. TSPM degrades directionally toward the smooth surface direction and facilitates the sustained release of small molecules or biological growth factors directionally into the defect. We validated this technology in a rat periodontal defect model to enable local regeneration as proof‐of‐principle. Overall, this periodontal membrane technology presents a versatile strategy for facilitating localized tissue regeneration and directed therapeutic release, overcoming shape maintenance challenges of current clinical materials through a novel chemical approach.

## 2 Results

### 2.1 Nanofiber Formation in a Thermosensitive Matrix Combining Crystalline and Amorphous Components

**Figure 1:**
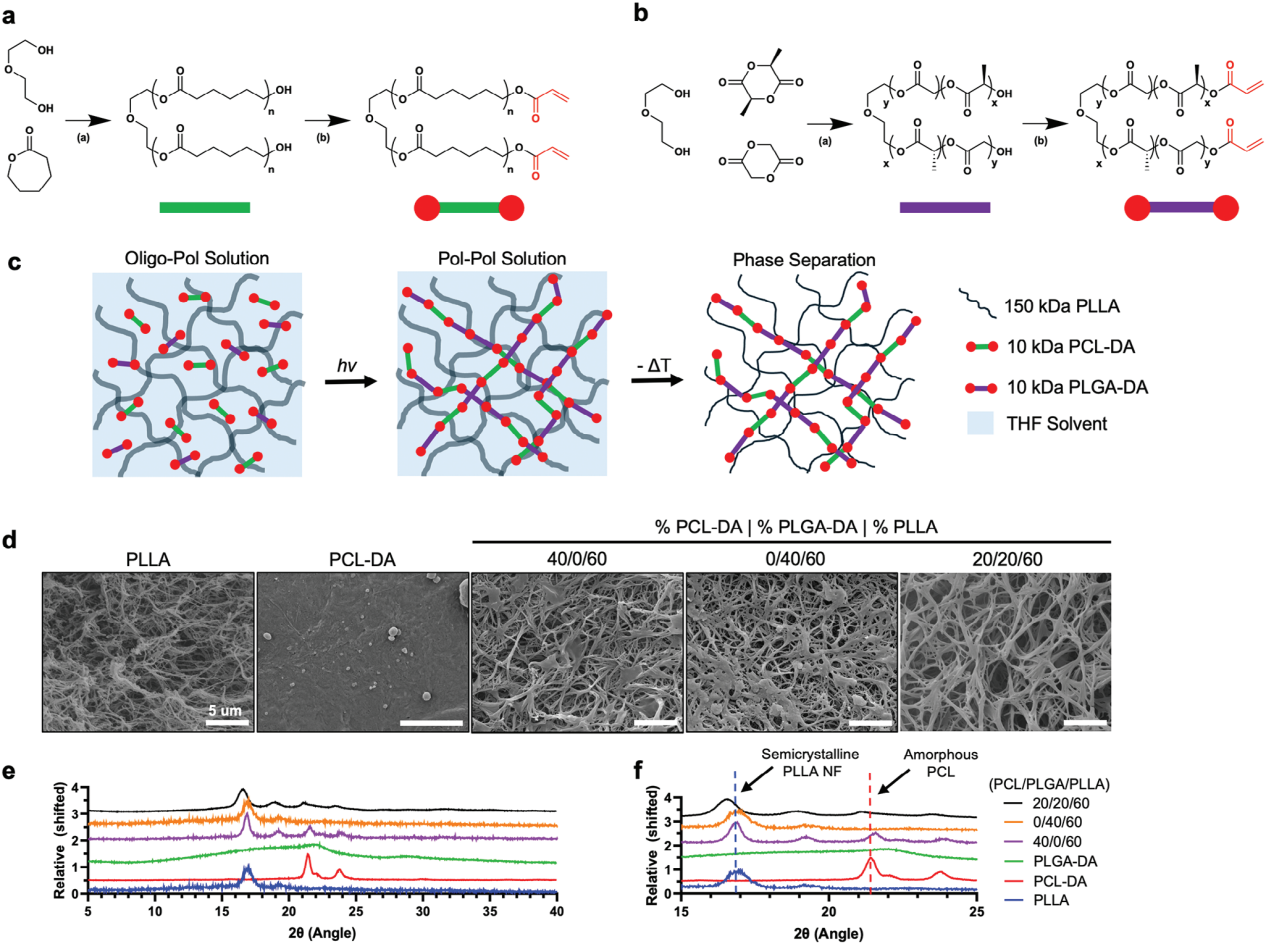
Polycaprolactone diacrylate (PCL‐DA, a) and poly (lactide‐s‐glycolide) diacrylate (PLGA‐DA, b) are synthesized by ring opening metastasis polymerization from 1,4‐butanol [a): 100710 125232 1,4‐butanol, Sn(Oct)_2_, vacuum, 120 °C, 4 h] and subsequently functionalized by nucleophilic substitution with acryloyl chloride [b): triethylamine cat., DCM solvent, 0 °C, 6 h]. A solution of poly (L‐lactic acid) (PLLA, 150 kDa) is combined with a solution of PCL‐DA (10 kDa) and PLGA‐DA (10 kDa) oligomers (oligo‐pol solution), including photoinitiator and UV‐irradiated to induce PCL‐DA—PLGA‐DA chain extension polymerization, in solution, shown schematically in C. Thermally‐induced phase separation (TIPS) causes the polymer‐rich phase to separate from the solution, resulting in nanofiber formation c). Nanofiber formation is assessed as a function of polymer composition by scanning electron microscopy (SEM, d, scale = 5 µm). Crystallinity is measured by small angle x‐ray diffraction (XRD; e, f).

We previously demonstrated a thermosensitive biomaterial with memorized microstructure may be fabricated by the in situ polymerization of a semicrystalline thermosensitive polyester mesh, polycaprolactone (PCL), within a rigid high molecular weight crystalline poly(L‐lactic acid) (PLLA) matrix and determined a critical threshold of 60% PLLA, under which nanofiber formation by thermally induced phase separation is compromised (Woodbury et al., *Frontiers in Dental Medicine*, 2023).^[^ ^38^ ^]^ We hypothesized that introducing poly lactide‐co‐glycolide (PLGA), an amorphous polymer, may favorably improve the bulk material properties due to its hydrophilicity, facilitating more rapid degradation, and amorphous nature, allowing it to serve as a thermosensitive chaperone in a similar capacity to PCL. PCL and PLGA macromers are synthesized from their respective monomers as ring‐opening metastasis polymerization; the terminal functional groups are acrylate‐modified by nucleophilic substitution with acryloyl chloride yielding PCL‐DA and PLGA‐DA (**Figure** **1**a,b) to allow for chain‐end extension reaction. In a solution of high molecular weight PLLA (150 kDa, Figure S1, Supporting Information), low molecular weight (≈10 kDa) PCL‐DA (Figure S2, Supporting Information), and PLGA‐DA (Figure S3, Supporting Information), macromers are polymerized within the PLLA mesh, yielding a semi‐interpenetrating matrix. Thermally‐induced phase separation (TIPS) induces nanofiber formation by the highly crystalline PLLA, yielding a nanofibrous PLLA matrix with interpenetrating thermosensitive PCL and PLGA components (Figure 1c). Compared to a 100% PLLA matrix and our previously described 40% PCL‐DA/60% PLLA matrix,^[^ ^38^ ^]^ the lower limit boundary condition for nanofiber formation, the introduction of PLGA‐DA into the PLLA matrix at up to 40% w/w allows for maintenance of nanofiber formation (Figure 1d). A 20% PCL‐DA/20% PLGA‐DA/60% PLLA composition (Figure S4, Supporting Information) facilitates uniform nanofiber formation, with fiber diameters ranging from 50–500 nm, miming the native collagen extracellular matrix. Small angle x‐ray scattering demonstrates the maintenance of PLLA crystallinity with characteristic peaks^[^ ^39^ ^]^ at 2*θ* = 17°, 19°, and PCL, 2*θ* = 22°, 24° which are preserved when fabricated as nanofibers and when combined with PCL‐DA at up to 40% PLGA‐DA/60% PLLA or 40% PCL‐DA/60% PLLA. PLGA does not show a significant x‐ray signature compared to PCL‐DA, confirming its amorphous nature (Figure 1e,f).

### 2.2 Self‐Assembling Bilayer Formation by Open‐Sandwich Fabrication Method

**Figure 2:**
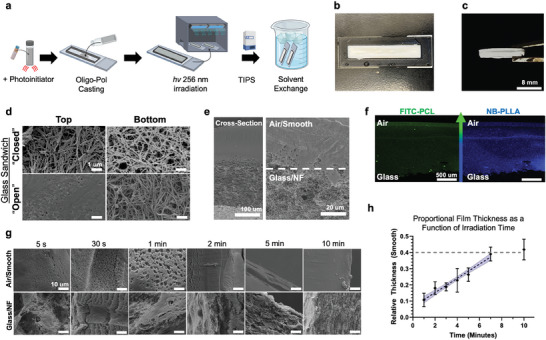
In situ polymerization is carried in a UV‐irradiation chamber (405 nm, a) in either an “open sandwich” (glass bottom with no glass covering) or a “closed sandwich” (glass bottom with glass slide covering) method yielding a uniform membrane b,c). Nanofiber formation in 20/20/60 membranes, as a function of the casting strategy, is evaluated by SEM (d, scale = 1 µm). A transverse section of an open sandwich 20/20/60 membrane demonstrates bilayer formation, visualized by SEM (c, scale = 50 µm) with distinct smooth and nanofibrous regions (e, left scale = 100 µm, right scale = 20 µm). Membranes were fabricated using fluorescently labeled PLLA (nile blue, blue) and PCL (FITC, green) and illustrate preferential partial phase separation within the bilayer (f, scale = 500 µm). The thickness and porosity of the air‐facing layer are visualized by SEM (g, scale = 10 µm) and measured as a function of irradiation time (h, n = 3 measurements per image, n > 4 images per sample, n > 4 samples per composition).

Membranes were fabricated using a solvent casting procedure and in situ polymerization. Membrane frames were 3D printed and adhered to glass substrates (Figure S5, Supporting Information). Following casting, the PLLA‐oligomer solution (e.g., 20% PCL‐DA, 20% PLGA‐DA, 60% PLLA in THF) was irradiated at 256 nm and then subjected to thermally induced phase separation (**Figure** **2**a), yielding a uniform bulk material (Figure 2b,c). At the casting step, covering the pre‐polymerized solution with glass (“closed sandwich”) resulted in a uniformly nanofibrous surface texture on both sides of the resulting membrane (Figure 2d). However, leaving the pre‐polymer solution uncovered during crosslinking (“open sandwich”) and then covering after irradiation resulted in a self‐assembled bilayer where the air‐facing side (top) is smooth, mimicking a PCL matrix.

In contrast, the glass‐facing (bottom) side is uniformly nanofibrous (Figure 2d). The cross‐section distinguishes between distinct yet continuous membrane segments with smooth or nanofibrous morphologies (Figure 2e), achieving a desirable bilayer membrane. To determine the distribution of the polymer components within the self‐assembled membrane, we synthesized fluorescently labeled PLLA (nile blue‐PLLA) and PCL (fluorescein isothiocyanate, FITC‐PCL) and fabricated a membrane from 40% PCL‐DA/60% PLLA, each containing 5% fluorescently labeled polymer as a dopant. Cross sections visualized by confocal laser microscopy demonstrate a nonhomogeneous macroscopic distribution of Nile blue‐PLLA and FITC‐PCL, although both polymers are present in the entire film (Figure 2f). FITC‐PCL localizes toward the air‐facing layer, while nile blue‐PLLA concentrates toward the glass‐facing layer. By scanning electron microscopy, we assayed bilayer formation as a function of irradiation time to determine the relative thickness of each in our fabrication protocol. The bilayer is apparent within 1 min of irradiation (256 nm, 10 J, Figure 2g, and its relative thickness continues to increase to ≈8 min (Figure 2h; Figure S6, Supporting Information). The overall thickness of the membrane may be modulated by altering the volume of the prepolymer solution and frame height while maintaining the biphasic morphology (Figure S7, Supporting Information).

### 2.3 PLGA Constituent Increases Hydrophilicity of the Matrix

**Figure 3:**
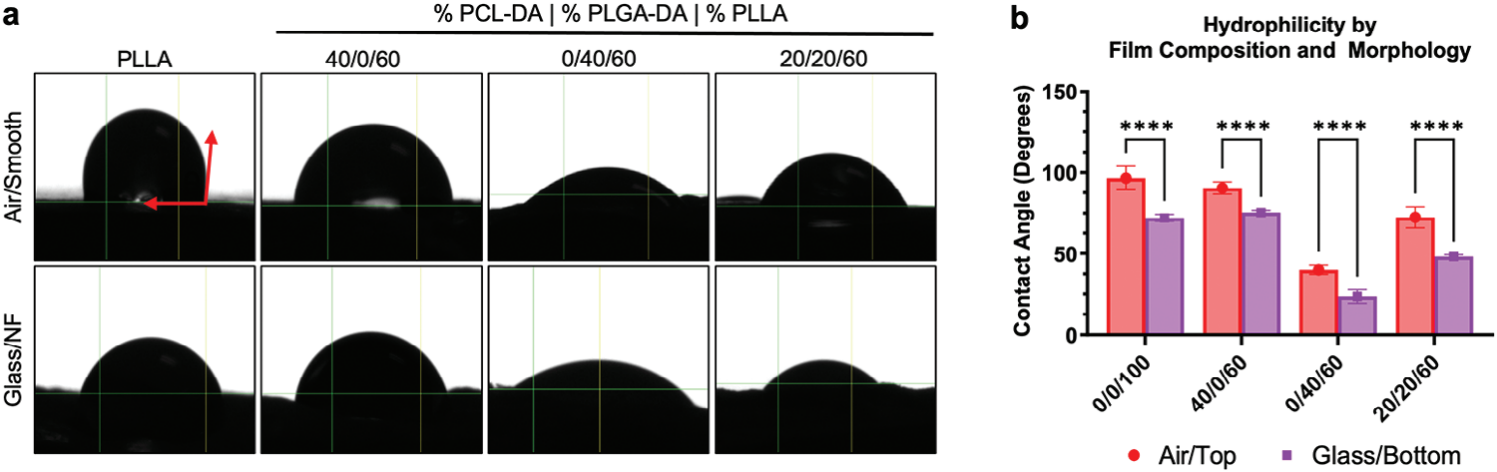
The contact angle with water of the air‐facing (smooth) and glass‐facing (nanofibrous) sides of membranes fabricated with the open sandwich technique is measured as a function of composition a,b). n = 3 images per sample, n = 4 samples per group. ****: *p* <  0.0001.

The wettability of a biomaterial's surface directly influences its biocompatibility by affecting cell adhesion, protein adsorption, and its ability to integrate with tissues, a key feature of nanofibers in promoting cell adhesion and osteogenic differentiation.^[^ ^40^, ^41^, ^42^ ^]^ We hypothesized that incorporating PLGA‐DA as a replacement for some part of the PCL‐DA component would improve the wettability of the membrane and increase its hydrophilicity. Representative goniometer images of water droplets on the smooth and nanofibrous sides of various membrane compositions are shown (**Figure** **3**a), and contact angle measurements are quantified (Figure 3b). The incorporation of PLGA‐DA (20% PCL‐DA/20% PLGA‐DA/60% PLLA, 0% PCL‐DA/40% PLGA‐DA/60% PLLA) significantly decreases the wettability of both the smooth and nanofibrous sides of the matrix. Interestingly, in all cases, the contact angle of the soft side is considerably more than the nanofibrous side, further supporting self‐assembly within the 3D bilayer construct. Interestingly, the addition of PLGA‐DA allows for titration of the contact angle, which could be tuned to a specific biological need. Based on its favorable composition, 20% PCL‐DA/20% PLGA‐DA/60% PLLA was chosen for subsequent biologic characterization.

### 2.4 Shape Control and Rigidity of Thermosensitive Periodontal Membranes

**Figure 4:**
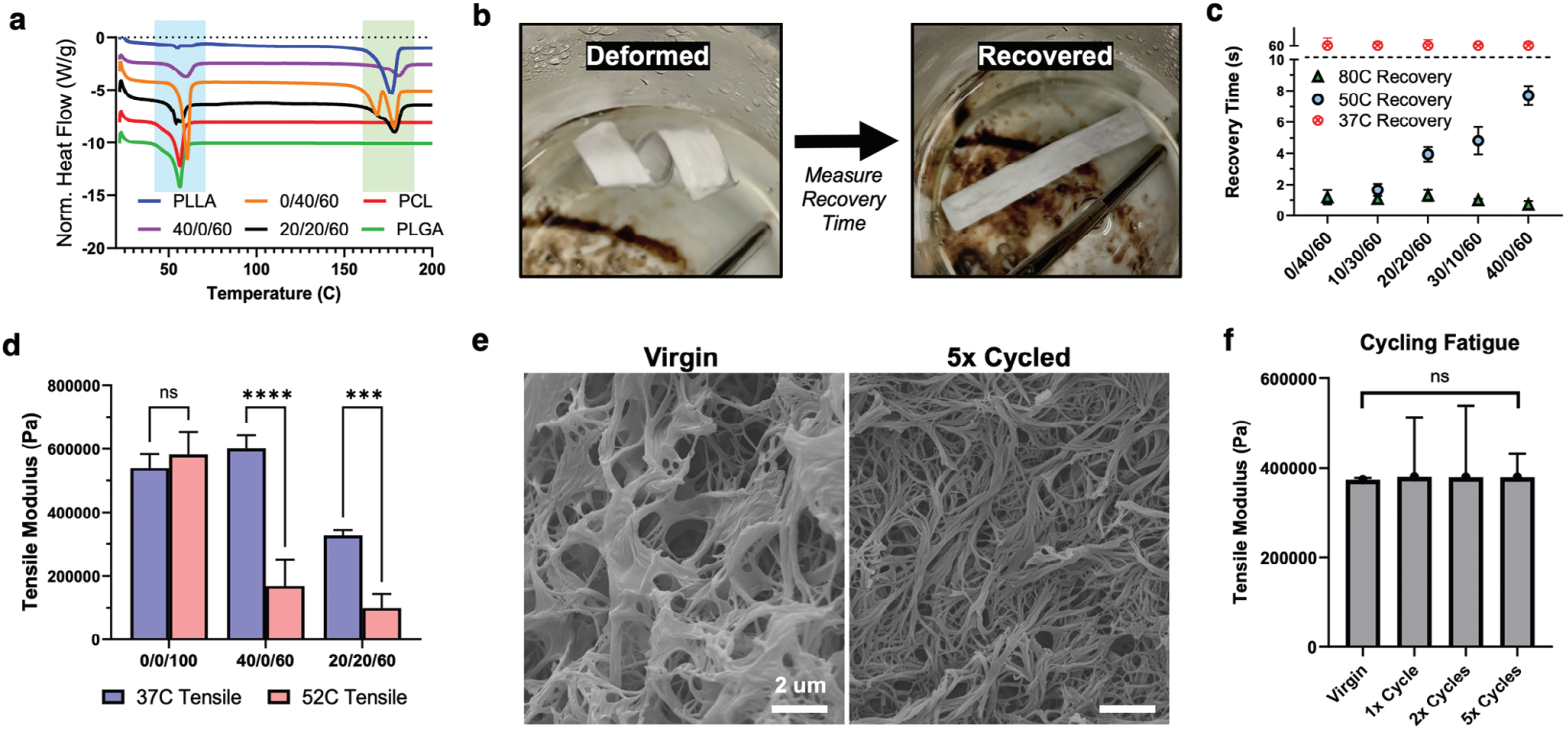
Membranes of various compositions were subjected to differential scanning calorimetry a) (DSC, n = 3 per group); two melting temperature ranges were identified at 50–55 °C (blue) and 160–170 °C (green). Membranes are heated in water baths at 52 °C, deformed and cooled. Deformed membranes are submerged in water baths at 50 or 80 °C; their recovery to the original (flat) shape is recorded by video b), and recovery time is measured by video analysis (c, n >  5 per group). Membrane mechanical properties are measured as a function of temperature and composition to determine the tensile modulus (d, n >  5 per group). Membranes are serially cycled through deformation‐recovery cycles up to five times. SEM evaluates nanofibers to compare virgin films to films cycled five times (e, scale = 2 µm). After each cycle, tensile modulus is measured (f. *: *p* < 0.05; **: *p* < 0.01; ***: *p* < 0.001; ****: *p* < 0.0001; n.s. = not significant.

Dynamic scanning calorimetry is used to quantify the temperature‐sensitive thermal properties of the TSPM. We observed a marked reduction in T_1_ melting temperature (T_m_) from 165 to 52 °C upon incorporating PCL‐DA (**Figure** **4**a). The incorporation of PLGA shifts T_1_ slightly lower, to 50 °C. Two distinct melting temperatures are observed in all compositions containing PCL‐DA and PLGA‐DA, where T_1_ corresponds to the thermosensitive chaperone component(s), and T_2_ exists at 165 °C, corresponding to the PLLA component. We confirmed the two‐component nature of our system and determined its degradation kinetics compared to PLLA and PCL‐DA by thermogravimetric analysis. We hypothesized that PLGA‐DA‐containing membranes would be deformable at or above T_m_ and able to recover their original morphology at an elevated temperature but not below T_m_ (Figure 4b). Following deformation, membranes rapidly recover at 80 °C, significantly greater than T_m_, and more slowly at 50 °C, following a linear trend relative to the amount of PLGA‐DA in the matrix (Figure 4c). In all cases, no recovery was observed at temperatures below 50 °C, which indicates the shape memory capacity of the TSM at various compositions and physiologic temperature (Figure 4c). Significant modulation of mechanical properties is observed as a function of temperature (Figure 4d; Figure S8, Supporting Information). Compared to 37 °C, at 52 °C, the yield point for 40% PCL‐DA/0% PLGA‐DA/60% PLLA and 20% PCL‐DA/20% PLGA‐DA/60% PLLA decrease significantly, while elongation at break increases significantly. Tensile modulus decreases significantly at 52 °C but is recovered at 37 °C, compared to PLLA, which does not show a temperature‐responsive decrease in tensile properties. The incorporation of PLGA‐DA decreases the overall tensile strength of the membrane. However, the material remains sufficiently rigid to withstand regular forces in the oral microenvironment. We performed serial deformation and recovery cycles to simulate repeated deformations of the material that may be required in a surgical environment. After deformation and recovery, TSPM nanofibers are indistinguishable from virgin material, even after five cycles, with no decrease in tensile strength (Figure 4e,f).

### 2.5 Biphasic Morphology Lends to Directional Properties

**Figure 5:**
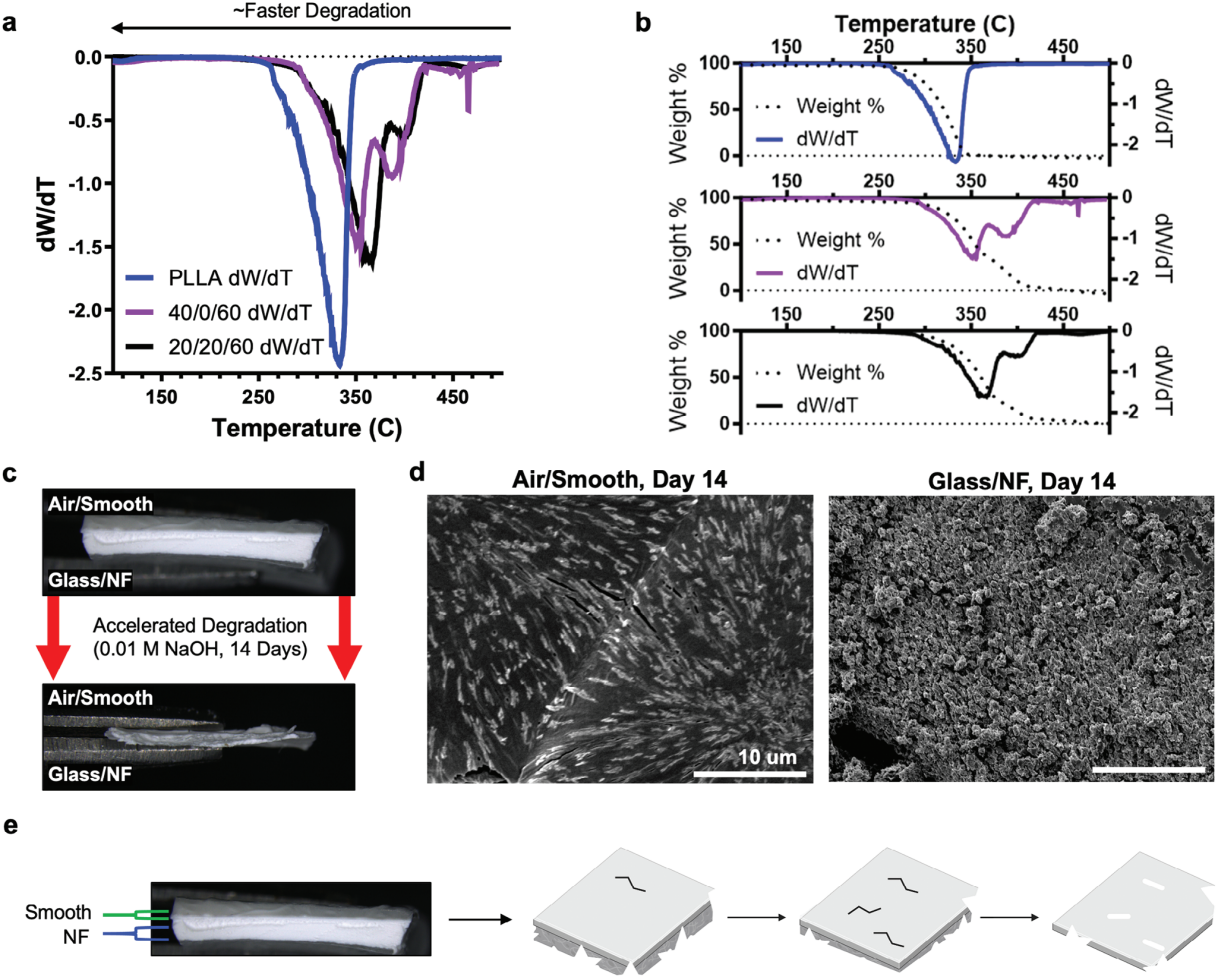
Thermogravimetric analysis is used as a proxy for the degradation profile of membranes as a function of polymer composition (a; b, dashed line = weight %, solid line = dW/dT). Gross images from an accelerated degradation test illustrate the directional degradation of the membrane from the nanofiber side (c). Membrane morphology after 14 days of accelerated degradation is assessed by SEM (d, scale = 10 µm) and shown schematically in e.

Given the apparent self‐assembling of PCL and PLLA domains within the TSPM and differences in surface area and wettability between smooth and nanofibrous textures, we hypothesized that our membranes may lend to several unique directional properties. First, we hypothesized a multicomponent degradation profile based on the different PLLA, PLGA, and PCL hydrophilicity. Thermogravimetric analysis confirms a multimodal degradation profile compared to PLLA (**Figure** **5**a). dW/dT represents a sample's mass loss rate as a function of time or temperature during the analysis. Adding PLGA shifts the degradation profile toward a lower temperature (toward the left), indicating accelerated degradation (Figure 5b). We subjected the 20% PLGA‐DA/20% PCL/60% PLLA TSPM to an accelerated degradation simulation (0.01 M NaOH, 14 days, 37 °C, Figure 5c). We demonstrated a directional degradation where the nanofibrous side of the membrane degrades more rapidly than the smooth side (Figure 5d), shown schematically in Figure 5e.

**Figure 6:**
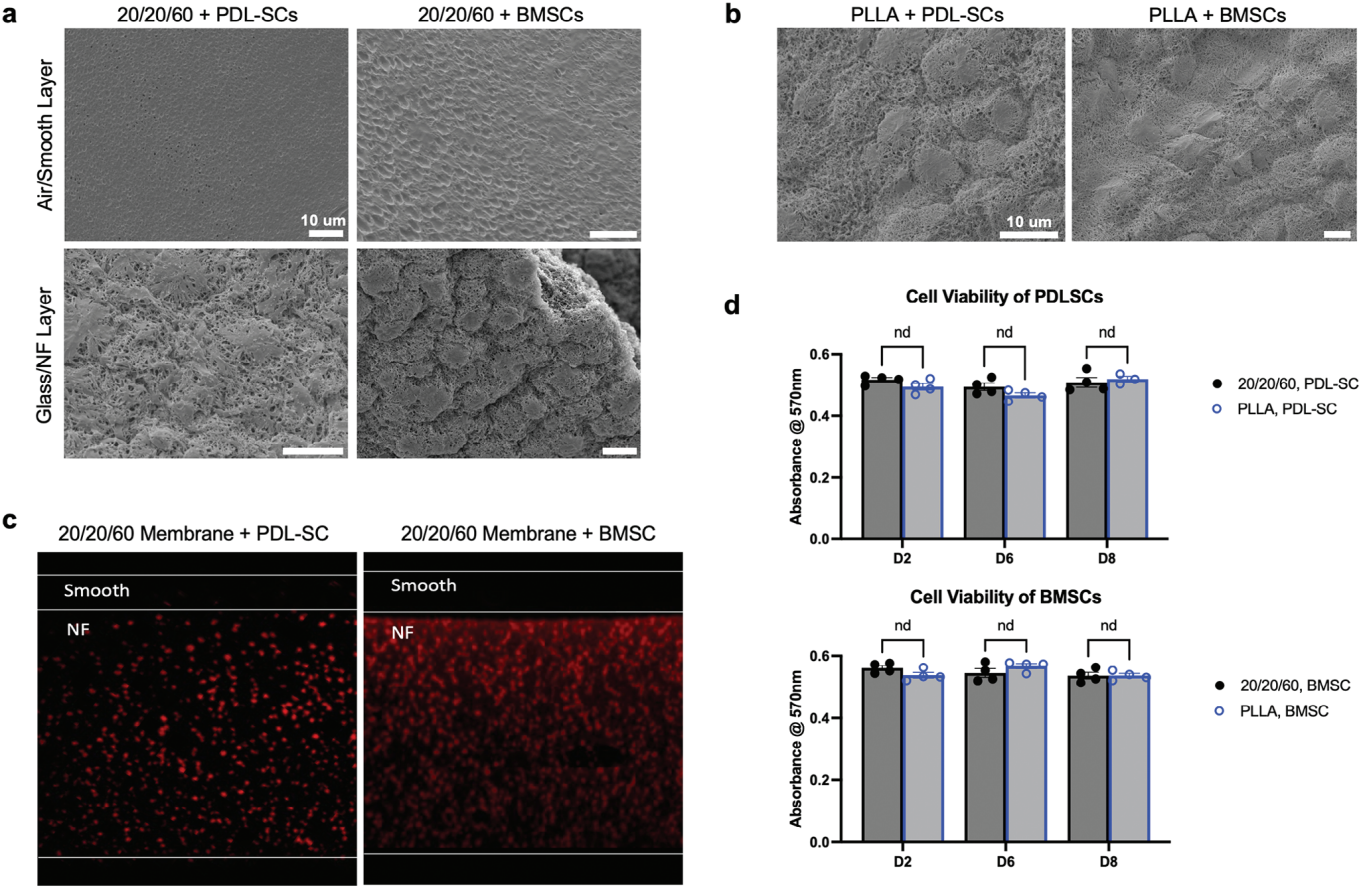
Periodontal ligament stem cells (PDL‐SCs) and bone marrow stromal cells (PDL‐SCs) are cultured on 20/20/60 membranes (a) or PLLA nanofibers (b) for eight days in vitro. SEM (scale = 10 µm) assesses cell morphology and surface distribution. Cell‐biomaterial constructs are fixed and sectioned, cells are fluorescently labeled (Phalloidin, red), and penetration into the construct is visualized by confocal laser microscopy (c, scale = 50 µm). Cell proliferation is assessed by MTA assay in vitro for up to 8 days (d).

The purpose of a bilayer membrane is to facilitate selective cell adsorption and infiltration, excluding epithelial ingrowth from outside of the defect while enabling regeneration inside of the defect. Human periodontal ligament stem cells (PDL‐SC) and mouse bone marrow stromal cells (BMSC) were cultured on the air‐inhibited smooth and nanofibrous sides of membranes from 20% PLGA‐DA/20% PCL/60% PLLA (TSPM composition used for all subsequent validation, **Figure** **6**a) or PLLA nanofibers (Figure 6b). Scanning electron microscopy demonstrates significant cellular adhesion to the nanofibrous, but not smooth, sides of the membrane after 24 h of culture (Figure 6a), comparable to PLLA nanofibers (Figure 6b). After eight days of culture, PLLA and TSMP cell‐biomaterial constructs were sectioned to observe cell infiltration and distribution throughout the depth of the construct. Visualized by confocal laser microscopy, both PDL‐SC and BMSC infiltrated from the nanofibrous side through the depth of the build but did not adhere nor migrate into the smooth region (Figure 6c). The PLLA and TSPM constructs maintained cell viability for up to 8 days, demonstrating biocompatibility (Figure 6d).

**Figure 7:**
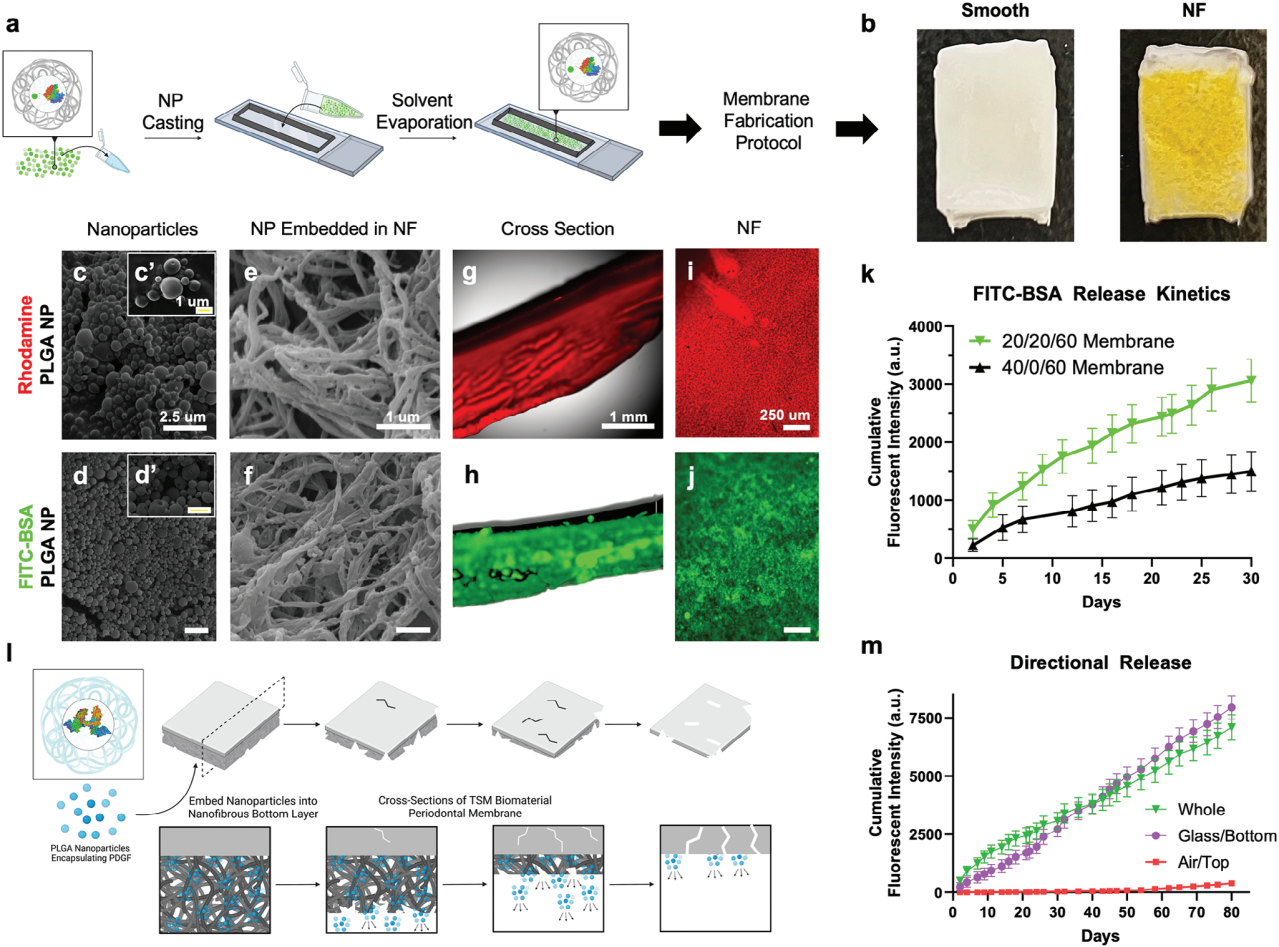
Nanoparticles are fabricated and drop cast to glass slides from an ethanol suspension (a), yielding membranes loaded with nanofiber‐embedded nanoparticles (b, yellow color from encapsulation of FITC‐BSA). PLGA nanoparticles encapsulating rhodamine (c, scale = 2.5 µm; C’, scale = 1 µm) or FITC‐bovine serum albumin (FITC‐BSA, d, scale = 2.5 µm; D’, scale = 1 µm) are fabricated by a double emulsion method. Lyophilized nanoparticles are suspended in an ethanol solution, then drop cast and dried to the membrane fabrication molds before casting the oligo‐pol solution. After fabrication, nanoparticles are encapsulated in the nanofiber (NF‐NP) as visualized by SEM (e, f; scale = 1 µm). Membranes are visualized by confocal laser microscopy to confirm the incorporation of fluorescent cargo, visualized in the transverse section (g, h; scale = 250 µm) and from the bottom (i, j; scale = 250 µm). Membranes are incubated in PBS at 37 °C; release kinetics are determined by measuring supernatant fluorescence (k, n >  3 per time point). Membranes were isolated to determine the directionality of sustained release, as shown schematically in (l), up to 80 days in vitro (m).

Previously, we described a novel nanofiber‐embedded controlled delivery system, where nanoparticles were introduced during the polymerization step and directly embedded into the nanofiber through nonspecific hydrophobic interaction rather than surface adhesion after fabrication.^[^ ^38^ ^]^ We developed a modified TSPM fabrication protocol whereby drug‐loaded particles were surface‐coated onto the glass substrate in a thin layer using ethanol as a dispersant and dried before TSMP fabrication (**Figure** **7**a,b). PLGA nanoparticles (NP, d_avg_ = 200 nm) were fabricated by a water‐in‐oil‐in‐water double emulsion, as described in the literature,^[^ ^43^ ^]^ containing Rhodamine B (RhB), a small molecule model drug, and fluorescein isothiocyanate bovine serum albumin (FITC‐BSA), a model protein therapeutic. Particles were lyophilized, and their morphology was assessed (Figure 7c,d). After in situ polymerization and TIPS, scanning electron microscopy confirms the physical incorporation of RhB‐NP and FITC‐BSA NPs into the nanofibers (Figure 7e,f); their regional specificity and incorporation are confirmed by confocal laser microscopy (Figure 7g‐j).

Further, we hypothesized that combining nanofiber‐specific delivery vector localization and directional degradation of the TSPM would enable the directional release of therapeutic cargo into the defect site, overcoming the limitations of the current lack of delivery technology. Given the relevance of biotherapeutics to periodontal regeneration, we assessed the sustained release of FITC‐BSA from the membrane under physiologic conditions (Figure 7k); the incorporation of the PLGA‐DA component significantly accelerates release on a time scale relevant to early osseous healing in the periodontal microenvironment. We specifically isolated various surfaces of TSMP loaded with FITC‐BSA NPs to determine the contribution of the smooth and nanofibrous sides of the membrane. The nanofiber compartment of the TSMP is solely responsible for the controlled release of therapeutic cargo, enabling directional release into the defect site (Figure 7l,m).

### 2.6 Evaluation in a Periodontal Defect Model In Vivo

**Figure 8:**
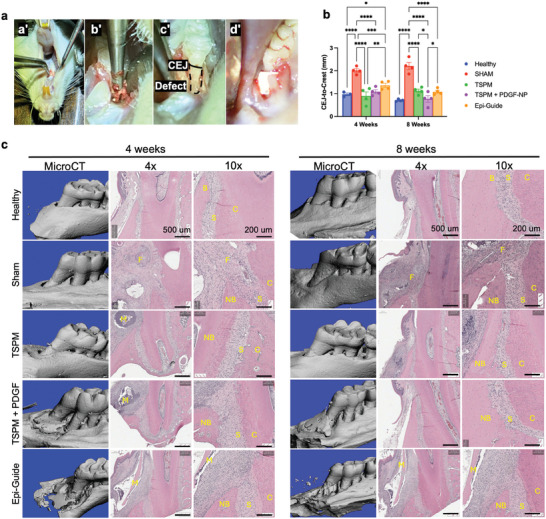
An osseous periodontal defect is made bilaterally in the maxillary first molar of rats, as shown in (a). Briefly, animals are anesthetized, and the area is scrubbed before administering local anesthesia in a restraint device designed for dental procedures. A full‐thickness incision is made on the medial aspect of the first molar to reflect the mucosa and expose the cementoenamel junction (CEJ) and alveolus. A ½ round (size) carbide bur is used to make a bony defect along the mesial root of the first molar, the size of the bur (b). Surgical debris is removed, and the exposed cementum is root planed using a microscaler (c). A periodontal membrane is placed at the defect site (d); mucosa is repositioned and stabilized using cyanoacrylate (d). Bone loss is quantified from MicroCT scans and shown in (b), with representative CT and histologic images at 4 and 8 weeks (c). *: *p* < 0.05; **: *p* < 0.01; ***: *p* < 0.001; ****: *p* < 0.0001; n.s. = not significant. C: cementum, S: Sharpey's fibers, NB: new alveolar bone, F: fibrous tissue, M: membrane.

We sought to validate the ability of the TSPM to facilitate guided tissue regeneration in vivo, compared to a commercially available synthetic bilayer membrane (Epi‐Guide/GUIDOR Bioresorbable Matrix Barrier). Adult rats were subjected to a bilateral osseous defect on the mesial root of the first molar, root planing, and treated with no membrane (sham), TSPM, TSPM + PDGF‐NP, Epi‐Guide, compared to untreated (healthy) rats (**Figure** **8**a; Figure S9, Supporting Information). At the terminal endpoint, 4 or 8 weeks after surgery, rats were euthanized, and the surgical site was evaluated by microcomputed tomography and histology (Figure 8b,c; Figure S9, Supporting Information). In all cases, no dehiscence was noted. After 4 and 8 weeks, negligible healing is observed in sham‐treated rats based on the distance between the alveolar bone and cementoenamel junction (CEJ; 4 weeks: 2.010 ± 0.160 mm vs 0.9700 ± 0.106 mm, *p* < 0.001; 8 weeks: 2.207 ± 0.313 mm vs 0.7050 ± 0.046 mm, *p* < 0.001). Significant inflammatory infiltration and soft tissue infill along the mesial root of the first molar are observed histologically, with no alveolar bone regeneration and disorganized Sharpey's fibers. Gingival inflammation persists for up to 8 weeks. Each of TSPM, TSPM + PDGF‐NP, and Epi‐Guide prevented epithelial ingrowth into the defects and promoted osseous wound healing at the defect site. Clinically and histologically, the gingiva healed well with an expected zone of attached masticatory mucosa surrounding the teeth, as visualized by H&E staining (Figure S10, Supporting Information). After 4 weeks, TSPM and TSPM + PDGF showed accelerated healing compared to Epi‐Guide (0.8975 ± 0.355 mm vs 1.084 mm ± 0.174 vs 1.363 ± 0.182 mm). By 8 weeks, the three membrane treatments showed favorable results between TSPM, TSPM + PDGF, and Epi‐Guide, while the addition of PDGF showed the most superior result (1.121 ± 0.150 mm vs 0.848 ± 0.341 mm vs 1.082 ± 0.132 mm). Histologically, osseous wound healing and new alveolar bone deposition are noted at 4 weeks and more dramatic after 8 weeks. The bone pattern is well organized, lamellar, and has a uniform and narrowing periodontal ligament space, indicative of inflammatory resolution. The Sharpey's fibers are well organized, and the periodontal ligament space is well‐cellularized in both TSPM and TSPM + PDGF‐treated rats after 4 weeks. Compared to Epi‐Guide‐treated rats, TSPM and TSPM+PDGF‐treated rats show a more organized interface between Sharpey's fibers and the cementum, which may indicate a more physiologic and matured periodontal ligament. The TSPM membranes demonstrated adequate degradation throughout the study, further supporting their compatibility and effectiveness within the biological context under investigation. We observed significant degradation of TSPM after 4 weeks, while the Epi‐Guide membrane persists even up to 8 weeks and is significantly slower to degrade.

## 3 Discussion

Herein, we have described a novel synthesis and fabrication of a thermosensitive periodontal membrane from biodegradable synthetic polymers. Its novel chemical composition combining PLLA, PCL‐DA, and PLGA‐DA in an optimized semi‐interpenetrating network yields a thermosensitive matrix pliable at elevated temperatures and rigid at physiologic temperatures. These properties lend themselves useful for personalized medicine where it is conceivable that the TSPM membrane could be heated and precisely contoured to each patient's oral defect. Cooling to physiological temperature maintains its deformed shape to maintain the dimensional fidelity of the periodontal defect, overcoming significant limitations of currently available guided tissue regeneration membranes. The self‐assembling biphasic nature, a product of our open‐sandwich fabrication technique and differential phase separation of PLLA and PCL allows for an outward‐facing smooth film, which we demonstrated to be impenetrable by cells and slower to degrade, compared to the nanofibrous side. The smooth side prevents epithelial ingrowth in a clinical defect. The inward‐facing nanofibrous mesh acts as a scaffold to facilitate cell and tissue ingrowth and differentiation and degrades concomitantly with bone remodeling. Importantly, we demonstrated the ability of cells to survive on and within the nanofibrous mesh and that thermal cycling and mechanical deformation do not disrupt this favorable surface topology, which supports tissue integration and bone formation previously described to occur through protein adsorption.^[^ ^40^ ^]^

PLLA has been widely used as a biocompatible material capable of nanofiber formation by thermally induced phase separation.^[^ ^33^, ^44^, ^45^, ^46^ ^]^ Nanofibers, like those we observe in the TSPM, have been commonly studied in PLA‐based materials. Their ability to promote cell adhesion, proliferation, and osteogenic differentiation is attributed to their high surface area, which adsorbs cell‐secreted proteins early in the regenerative process both in vitro and in vivo.^[^ ^40^, ^42^, ^47^ ^]^ We initially proposed a PCL‐PLLA composite where the PCL polymer component is interwoven in the PLLA mesh by a semi‐interpenetrating network to act as a thermosensitive chaperone of high‐molecular‐weight PLLA.^[^ ^38^ ^]^ While adequate for a temperature‐sensitive material by itself, the use of PCL as a significant component in tissue engineering applications has been stunted because of its inferior mechanical properties and long degradation time.^[^ ^33^, ^48^ ^]^ To overcome this challenge, we hypothesized that a PLGA‐PCL copolymer may be more advantageous in improving biocompatibility and favorable degradation kinetics because of the added hydrophilicity and lower half‐life of PLGA. Based on the TGA and DSC data presented, the addition of PLGA to PCL can decrease the average crystallinity of PCL, suggesting that the two polymers are miscible and can interact to form a blend with intermediate properties. The blend's properties, including crystallinity and hydrophilicity, can be controlled by adjusting the ratio of PCL to PLGA, which could be tailored to meet specific requirements for biomedical applications.

Similarly, while we used a consistent 50:50 lactide to glycolide ratio in PLGA, this ratio may be modulated to further refine the TSPM membranes properties. Our semi‐interpenetrating network design was considered advantageous over a fully interconnected three‐component polymer network because it provides macromolecular freedom of rearrangement by the PCL‐s‐PLGA within the PLLA matrix, which we attribute to be the fundamental molecular mechanism yielding temperature‐sensitive changes in mechanical properties in the TSPM compared to PLLA alone.^[^ ^49^ ^]^ Furthermore, nanofiber formation depends on the crystallinity of PLLA, which would not be possible in a statistical copolymer polymer network of PLLA, PCL, and PLGA.^[^ ^44^ ^]^

A key feature of our TSPM platform is its ability to spontaneously form a continuous bilayer within a single construct—a product of the “open sandwich” fabrication technique, which is a simple yet elegant strategy taking advantage of differential chemical properties of the system components. Glass substrates typically have high surface energy, characterized by their ability to form hydrogen bonds and engage in polar interactions. A significant concern with existing collagen bilayer membranes is the potential for delamination between the layers, which can compromise the membrane's structural integrity and functional performance. Delamination may occur due to inadequate bonding between the layers, differences in mechanical properties, or degradation rates of the materials used in each layer.^[^ ^50^, ^51^, ^52^ ^]^ This can lead to membrane failure, inadequate tissue regeneration, or the need for additional surgical interventions. A single construct with a continuous bilayer is advantageous to clinicians as it can offer seamless integration between the layers, reducing the risk of delamination and improving the membrane's overall mechanical strength and stability.

The design of our TSPM bilayer membrane with differential degradation rates between the nanofibrous defect‐facing side and the epithelial‐facing smooth side is advantageous for periodontal regeneration for several reasons.^[^ ^53^, ^54^ ^]^ The faster degradation of the nanofibrous defect‐facing side allows for the ingrowth of cells and tissues necessary for periodontal regeneration. This side of the membrane is in contact with the defect and thus should support the regeneration of the periodontal ligament and alveolar bone, as we observed in the orthotopic rat model. As the nanofibrous side degrades, it allows for the gradual transfer of mechanical loads to the regenerating tissue, which can help develop a functional periodontal ligament and alveolar bone.^[^ ^55^ ^]^ The degradation of the nanofibrous side allows for remodeling of the scaffold as the new tissue forms, which can help create a more natural architecture of the regenerated periodontium, mimicking the structure and function of the native tissue.

Further, the bilayer enables directionally controlled sustained release of small molecule or biologic therapeutics by a nanofiber‐embedded nanoparticle delivery system. These membranes are designed to deliver therapeutics directly to the defect site by embedding nanoparticles homogeneously throughout the nanofibrous architecture, optimizing the regenerative process while minimizing exposure to surrounding tissues like the epithelium or gingiva. We demonstrate the linear sustained release of small molecule and protein cargo for up to 80 days, consistent with human healing time frames, avoiding the burst release observed in comparable literature.^[^ ^56^, ^57^ ^]^ This approach ensures that the growth factor is controllably administered and utilized where most needed, reducing waste and potentially lowering the PDGF required to achieve therapeutic effects. This targeted approach can make treatments more affordable and accessible.

In vivo, we demonstrated the clinical utility of the TSPM to facilitate robust alveolar bone regeneration in a localized periodontal defect model and regeneration of the periodontal ligament, including well‐organized Sharpeys fibers with adequate PDL space cellularization. Regenerating these fundamental structures is crucial in periodontal‐guided tissue regeneration because they are essential for re‐establishing the robust and functional attachment between the tooth and the surrounding alveolar bone. This restoration stabilizes the tooth and helps maintain the integrity and health of the periodontium, thereby preventing further periodontal disease and tooth loss. While a molecular characterization of the periodontal ligament and periodontal ligament stem cell niche is beyond the scope of the current investigation, it is an interesting perspective to consider. In this model, the first molar defect does not heal spontaneously; therefore, a biomaterial is required. Importantly, in the TSPM groups, no adverse effects were noted intraoperatively or following the surgery. The TSPM, with and without the controlled release of PDGF, facilitated alveolar bone regeneration based on microcomputed tomography and histologic results. Significantly, the membrane degrades on a physiologically relevant time scale, and minimal inflammation is observed. The Epi‐Guide PLLA membrane facilitates modest regeneration with more significant inflammation, less organized periodontal ligament space, and a slow degradation time.

Ultimately, our TSPM represents multiple advantages to enhancing synthetic membranes clinical performance and tissue regeneration capacity intraorally. First, we demonstrated the thermally‐induced change in mechanical properties due to the temperature‐sensitive PCL‐PLGA component. Above the critical temperature of 52 °C, the membrane is readily deformed to the shape of an alveolar ridge or defined defect. For example, a clinician may use a stone model or 3D printed cast of a patient, taken from an intraoral scan or cone beam computed tomography (CBCT) scan to perform the membrane to the patient's need. Once cooled to physiologic temperature, the membrane is rigid. This process can be repeated multiple times to adjust the shape to fit the clinical need. Biologically, the TSPM degrades from the nanofibrous (defect‐facing) side toward the smooth (epithelium‐occluding) side while directionally releasing biologic growth factors or small molecule therapeutics into the defect site, precisely defining and regulating the local regenerative process. The nanofiber‐embedded delivery system is highly modular, demonstrating linear controlled release for up to 80 days within the regenerative compartment. We demonstrate the capacity of the nanofibrous side, but not the smooth side, to facilitate the adhesion and proliferation of mesenchymal stromal cell populations relevant to periodontal regeneration. Compared to the commonly used collagen matrices, our material provides superior clinical handling properties in its ability to maintain its 3D shape without requiring tenting screws or a titanium mesh, which may act as a foreign body, poses a risk for dehiscence, and requires a second‐stage surgery for its removal. Regarding guided tissue or bone regeneration, the TSPM technology has significant potential for a breadth of surgical periodontal interventions.

## 4 Conclusion

In conclusion, we developed a semi‐interpenetrating network of PCL‐DA and PLGA‐DA oligomers, serving as a temperature‐sensitive chaperone that interpenetrates high molecular weight PLLA as a novel thermosensitive periodontal membrane (TSPM) which acts both as a barrier membrane and scaffold matrix. A minimum threshold of PLLA is required for nanofiber formation at 60% w/w. Incorporating PLGA into the PLLA/PCL composite not only improved the degradation properties of the membrane but also enhanced its mechanical handling properties, allowing for both deformation and rigid shape maintenance controlled by a thermosensitive chemical chaperone. The ratio of PCL‐DA to PLGA‐DA modulates contact angle, degradation, and release kinetics from an embedded delivery system. We identified 20% PCL‐DA/20% PLGA‐DA/60% PLLA as the ideal composition to maintain mechanical rigidity and thermoresponsive properties while having favorable biologic properties. This composition enables the formation of robust nanofibers with average diameters of 50–500 nm, with temperature‐sensitive mechanical properties allowing for reversible bulk deformation above 50 °C but rigid shape maintenance at physiologic temperature. We also demonstrated a novel method for incorporating nanoparticles into the membrane nanofibers, allowing directional sustained drug delivery specifically to the defect. This TSPM technology, both a membrane and a scaffold, holds promise for periodontal tissue engineering applications requiring vertical and horizontal bone augmentation and guided tissue regeneration due to its thermosensitive favorable handling properties to contain and augment periodontal defects. Future research should optimize the delivery system for growth factors and other biotherapeutics to maximize the regenerative outcomes. Additionally, exploring the applicability of this technology in different tissue engineering contexts could broaden its clinical utility.

## 5 Experimental Section

### Materials

Resomer 207S poly (L‐lactic acid) (PLLA) was purchased from Evonik. Unless mentioned otherwise, all other reagents were purchased from Sigma–Aldrich. Reagents were used as received unless otherwise specified.

### Synthesis of Polycaprolactone Diol (PCL)

To prepare poly ε‐caprolactone (MW = ≈10 kDa), 10 mL of ε‐caprolactone monomer was added to a 50 mL round‐bottom flask, along with 89 µL of 1,4‐butanediol (1 mol eq). Next, 6.5 µL (0.01 mol eq) of tin(II) 2‐ethylhexanoate (Sn(Oct)_2_) was added to the flask, which contained a magnetic stir bar. The mixture was then stirred at a low speed under vacuum. The flask was heated to 120 °C and maintained for 12 h, resulting in a highly viscous solution. After cooling, the solid was dissolved in dichloromethane (DCM) and precipitated into 300 mL of methanol (≈5x volume) at 0 °C. This process yielded a white solid, which was then concentrated. Precipitation was repeated three times to remove any unreacted monomer and catalyst. The resulting solid was dried in a vacuum chamber for two days and stored at −20 °C. Finally, molecular weight was assessed using gel permeation chromatography (GPC) in tetrahydrofuran (THF) solvent, and molecular characterization was performed by nuclear magnetic resonance spectroscopy (NMR, CDCl_3_): 𝝳 = m, 1.48 (2H, CH_2_); m,1.78 ppm (4H, CH_2_); q, 2.44 ppm (2H, CH_2_); d, 4.17 ppm (2H, CH_2_).

### Synthesis of Polycaprolactone Diacrylate (PCL‐DA)

5 g of PCL‐diol (MW_avg_ = 10.47 kDa) was dissolved in a minimum volume of anhydrous DCM. 135 µL of triethylamine (TEA, 2 mol eq.) was added with stirring at moderate speed at 0 °C. After 15 min, 78 µL of acryloyl chloride (AC) was added dropwise into the solution over 10 min. The reaction was left to proceed overnight, warming to room temperature. The resulting reaction mixture was precipitated into 300 mL of methanol (≈5x volume) at 0 °C three times to remove unreacted AC and TEA. The concentrated solid was dried in a vacuum chamber for 2 days and stored at −20 °C. End‐group functionalization was confirmed by NMR (CDCl_3_): 𝝳 = m, 1.48 ppm (2H, CH_2_); m,1.78 ppm (4H, CH_2_); q, 2.44 ppm (2H, CH_2_); d, 4.17 ppm (2H, CH_2_); dd, 5.8 ppm (CH); m, 6.15 ppm (CH); dd, 6.44 ppm (CH).

### Synthesis of poly (lactide‐s‐glycolide) diol (PLGA)

Lactide (7.6 mmol, 1.10 g, 1 mol eq), glycolide (7.6 mmol, 0.88 g, 1 mol eq), and benzyl alcohol (0.2 mmol, 208 µL, 0.027 mol eq) were mixed and heated at 120 °C under vacuum with moderate stirring. Then, 100 µL of Sn(Oct)_2_ (0.001 mol eq) catalyst was added. After 12 h, the reaction was cooled and opened to air. The solid was dissolved entirely in DCM, and the resulting solution was precipitated into 300 mL of 0 °C methanol (≈10x volume) three times, resulting in a white solid. The solid was dried for two days in a vacuum chamber. Chemical identity and the lactide‐glycolide ratio were confirmed by NMR (CDCl_3_): 𝝳 = m, 4.75 ppm (2H, CH_2_). GPC determined molecular weight (9.89 kDa)

### Synthesis of poly (lactide‐s‐glycolide) diacrylate (PLGA‐DA)

9.89 kDa of PLGA‐diol was dissolved in a small amount of anhydrous DCM. 2 mol eq. of triethylamine (TEA) was added with moderate stirring at 0 °C. Then, 78 µL of acryloyl chloride (AC) was added dropwise into the solution for 10 min. The reaction mixture was allowed to proceed overnight and warm to room temperature. The mixture was precipitated into 300 mL of methanol (≈5x vol) at 0 °C to remove unreacted AC and TEA thrice. The concentrated solid was then dried in a vacuum chamber for two days and stored at −20 °C. End‐group functionalization was confirmed by NMR (CDCl_3_): 𝝳 = m, 4.75 ppm (2H, CH_2_); dd, 5.8 ppm (CH); m, 6.15 ppm (CH); dd, 6.44 ppm (CH).

### Nuclear Magnetic Resonance Spectroscopy

Spectra were recorded using a Varian spectrometer operating at 400 MHz and room temperature. Spectral analysis was performed using MestReNova (Version 12.0.0‐2000080, Metrelab Research).

### Gel Permeation Chromatography

A Shimadzu GPC system with a refractive index and diode array UV–vis detector was used to run the samples with THF solvent. The samples were prepared at 5 mg mL^−1^ and filtered before analysis.

### Thin Film Fabrication

The solution was quickly poured onto a 5‐inch silicon wafer and placed in a FisherScientific UV Crosslinking Chamber (λ = 256 nm) powered at E = 10 J for 5 min. The wafer was then encased in glass and immediately transferred to −80 °C for 48 h. After 2 days, the organic solvent was replaced with water at room temperature for 4 h. Finally, the resulting films were dried flat for 4 days, lyophilized, and evaluated for nanofiber formation.

### Scanning Electron Microscopy

The sample's morphology was studied using scanning electron microscopy (JEOL JSM‐7800 FLM) at an accelerating voltage of 5 kV and a working distance of 10–15 mm. Before observation, the samples were coated with gold using a sputter coater (Desk II, Denton Vacuum Inc.).

### Small‐Angle X‐Ray Scattering

A Rigaku Ultima IV Diffractometer obtains SAXS spectra from solid‐phase samples. The X‐ray was generated from a 2.2 kW Cu K‐alpha radiation long‐fine focus tube (0.4 × 12 mm) with cross‐beam optics. The scans were conducted with a Theta/Theta wide‐angle goniometer within the range of −3° to +154° (2*θ*) at a speed of 1° per minute. The D/teX‐ULTRA high‐speed detector detects the signal.

### Membrane Fabrication and In Situ Polymerization

TinkerCAD online software was used to design rectangular casting molds, for example, with an outline of 73 × 23 × 2 mm dimensions containing a central hollow rectangle of 60 × 10 × 2 mm. All casting molds were 3D printed from PLLA using an Original Prusa i3 MK3S+ 3D Printer. The 3D printed mold outlines were glued to the charged side of ASi SupremeTM Plus Microscope Slides using Gorilla Glue Super Glue and allowed to dry in a ventilated hood for 2 days before polymer casting.

In general, membrane molds for polymer casting were placed in a FisherScientific UV Crosslinking Chamber (λ = 256 nm) powered at E = 10 J. A 3 mM stock solution of Irgacure 2959 photoinitiator was prepared in methanol and stored at −20 °C. Separately, a 10% w/v polymer solution in tetrahydrofuran (THF) was prepared from varying w/w/w ratios of PLGA‐DA, PCL‐DA, and PLLA, respectively. The polymer solution was heated to 62 °C until the polymers were dissolved entirely. Once the polymer solution was dissolved, an aliquot of the photoinitiator stock solution (3.33% v/v) was added using a needle and syringe and mixed before casting and crosslinking in the UV chamber. After the time had elapsed, the membrane molds were removed from the UV crosslinking chamber and rapidly transferred onto dry ice. After 5–10 min, the membranes were moved into a −80 °C freezer.

After 48 h at −80 C, the membranes were removed from the freezer and placed into an ice bath. They soaked for ≈3 h while gradually warming to room temperature in the bath to remove organic solvent, turning bright white. The membranes were removed from the water after warming to room temperature, and the polymer membrane construct was removed. The resulting membranes were dried flat for 4 days and stored at −20 C until further use.

### Synthesis of Nile Blue PLLA (NB‐PLLA)

Nile blue‐PLLA was prepared using our previous method.^[^ ^58^ ^]^ Briefly, acrylic‐end functionalized PLLA was synthesized from hydroxyethyl methacrylate (HEMA) initiator (0.4 mmol, 100 µL) and L‐lactide (34.7 mmol, 5.00 g), with Sn(Oct)_2_ (120 µL) in a ring opening polymerization reaction (ROP) at 120 °C, in an inert nitrogen environment. After 12 h, the resulting HEMA‐PLLA polymer was exposed to air, cooled, dissolved in 20 mL chloroform, and precipitated in 100 mL cold methanol, and the product was collected. End‐group functionalization was confirmed by NMR (CDCl3) and FTIR. HEMA‐PLLA (1.40 g), nile blue acrylamide (0.012 mmol, 0.005 g), and freshly recrystallized AIBN (0.06 mmol, 0.0098 g, recrystallized from methanol) were dissolved in 10 mL dioxane at 70 °C and allowed to react overnight. The solvent was removed by rotary evaporation, and the resulting residue was dissolved in a minimum amount of chloroform and precipitated into cold methanol, then collected by suction filtration.

### Synthesis of FITC‐PCL

FITC‐PCL was prepared according to a modification of our previous method.^[^ ^58^ ^]^ Briefly, acrylic‐end functionalized PCL was synthesized from hydroxyethyl methacrylate (HEMA) initiator (0.4 mmol, 100 µL) and ε‐caprolactone (44 mmol, 5.00 g), with Sn(Oct)_2_ (120 µL) in a ring opening polymerization reaction (ROP) at 120 °C, in an inert nitrogen environment. After 12 h, the resulting HEMA‐PCL polymer was exposed to air, cooled, dissolved in 20 mL chloroform, and precipitated in 100 mL cold methanol, and the product was collected. End‐group functionalization was confirmed by NMR (CDCl3) and FTIR. HEMA‐PCL (1.40 g), FITC‐o‐acrylate (0.012 mmol, 0.005 g), and freshly recrystallized AIBN (0.06 mmol, 0.0098 g, recrystallized from methanol) were dissolved in 10 mL dioxane at 70 °C and allowed to react overnight. The solvent was removed by rotary evaporation, and the resulting residue was dissolved in a minimum amount of chloroform and precipitated into cold methanol, then collected by suction filtration.

### Confocal Laser Microscopy

A Nikon Eclipse C1 microscope was used for all confocal imaging.

### Contact Angle Measurement

The contact angle was measured using a Ramé‐Hart 200‐F1 goniometer. A small volume of liquid (2 µL) was advanced or receding onto the surface using a 2 mL micrometer syringe (Gilmont). At least six measurements were performed on each surface.

### Shape Memory and Recovery

Membranes were placed in a 50 °C water bath (regulated with a digital temperature probe) and submerged for 30 s. After this time, the membranes were coiled around a 1 mm diameter metal rod and transferred into an ice bath. Finally, the rigid membrane was transferred back into a 50 or 80 °C water bath. Membrane recovery was recorded using video and a digital camera. The videos were then analyzed to measure recovery time, n > 4 for each group.

### Differential Scanning Calorimetry (DSC)

To determine materials thermal and melting properties, a TA Instruments Discovery DSC was used according to protocols developed by the manufacturer with a scanning rate of 5 °C min^−1^. Each material was kept in aluminum pans, and an empty pan was used as the reference. All analyses were carried out in triplicate.

### Mechanical Testing

The tensile properties of membranes were measured using an MTS Synergie 200 mechanical tester (MTS Systems, Inc.). 60 × 10 × 1 mm membranes were prepared, n >  5 per composition and temperature. Tensile modulus was defined as the slope of the linear range on the resulting stress–strain curve, with a strain rate of 1.0 mm min^−1^.

### Thermogravimetric Analysis (TGA)

To determine materials thermal and melting properties, a TA Instruments Discovery TGA was used according to protocols developed by the manufacturer with a scanning rate of 20 °C min^−1^ and with inert gas flow. All analyses were carried out in triplicate.

### Fabrication of PLGA Nanoparticles

Nanoparticles were prepared using a w/o/w double emulsion method.^[^ ^57^ ^]^ In the case of RhodamineB, a model drug, 15 mg of RhodamineB was dissolved in 500 µL of distilled water (ddH_2_O). Separately, 250 mg of PLGA (50:50, MW = 7–17 kDa, Sigma) was dissolved in 1.50 mL of DCM in a 50 mL Falcon Tube. Both solutions were kept on ice. 160 µL of the drug solution was added to the PLGA solution and sonicated by a probe sonicator for 35 s on ice to create a w/o emulsion (35 V power). The w/o solution was poured into 5 mL of 1% w/v polyvinyl alcohol (PVA) in distilled water and sonicated for 35 s on ice. The w/o/w emulsion was transferred to a 20 mL glass vial and stirred at 1300 rpm overnight in a fume hood to allow for solvent evaporation. The nanoparticles were concentrated and washed by centrifugation at 9000 rpm for 20 min, reconstituting in fresh H_2_O between washes. Particles were reconstituted in ddH2O, lyophilized, and stored at −20 °C. In the case of FITC‐bovine serum albumin (BSA), a 1% w/v FITC‐BSA solution was prepared in PBS. 225 mg of PLGA (50:50, MW = 7–17 kDa, Sigma) was dissolved in 1.80 mL of DCM in a 50 mL Falcon Tube. Both solutions were kept on ice once fully dissolved, then 320 µL of the protein solution was added to the PLGA solution, and particles were fabricated as described.

### Fabrication of Nanofiber‐Embedded Nanoparticle (NF‐NP) Membrane

Membrane molds were 3D printed and glued to glass microscope slides. Slides were placed on a flat surface in a ventilated hood. A known mass of lyophilized particles (15 mg) was loaded into an Eppendorf microtube and suspended in 500 µL of ethanol (EtOH). The solution was transferred to a membrane mold to coat the glass surface nanoparticles. The solvent evaporated completely, leaving a nanoparticle‐coated substrate in the membrane mold. Once this process was complete and the nanoparticle‐coated membrane molds were dry, the fabrication method was identical to that described above.

### Controlled Release Kinetic Analysis

Membranes of known mass were incubated with PBS in a well plate at 37 °C on an orbital shaker. Fluorescence spectroscopy was used to measure the concentration of released rhodamine B and FITC‐BSA at various time points, and a Thermo ScientificTM VarioskanTM LUX Multimode Microplate Reader was used to quantify the fluorescence. The PBS solution from each well was removed at each time point, and the same volume of fresh PBS was added each time.

### Directional Release Kinetic Analysis

Membranes containing PLGA nanoparticles were cut into 13 × 10 × 1 mm rectangles. Cyanoacrylate glue was used to either invest the nanofibrous membrane side or the smooth membrane side to prevent its exposure and subsequent drug release, isolating the degradation and release to only the non‐glued part of the membrane. Carefully, Gorilla Super Glue was thickly painted onto the membrane slices nanofibrous parts, including the bottom face and four lateral sides. Other samples received the same treatment, but only to the top, smooth face of the membrane. The final group of membranes served as the control as they were untreated, leaving all surfaces exposed, and placed individually into wells of a 48‐well culture plate. The 48‐well plate was placed in a ventilated hood for 48 h to allow the super glue to dry. After this time, the experiment was carried out identically to the previous method for evaluating general drug‐release kinetics from the membranes.

### Sterilization of Biomaterial Constructs

First, constructs were sterilized by ethylene oxide gas according to the manufacturer's protocol (Anpro). Second, scaffolds were washed with 70% ethanol for 30 min, followed by PBS in a biosafety cabinet immediately before implantation.

### Cell Isolation

Primary bone marrow stromal cells (BMSCs) were isolated from femora and tibiae of 3‐week‐old mice and then cultured in Dulbecco's Modified Eagle Medium (DMEM) containing 10% FBS. All cells were used for experiments before passage 4. These procedures followed standards set forth and approved by the University of Michigan Institutional Animal Care and Use Committee (IACUC, PRO00011263). Human periodontal ligament (hPDL) tissues were harvested from the tooth root surface (IRB, HUM00142680).^[^ ^59^ ^]^ Briefly, freshly obtained tissues were cultured on alpha‐minimum essential medium (*α*‐MEM), then the PDL cells were collected, centrifuged, and resuspended at 37 °C for 60 min in a solution containing PBS, 2 mg mL^−1^ collagenase type II and 4 mg mL^−1^ dispase II. The mixture was inactivated using *α*‐MEM containing FBS and 100 µM ascorbic acid. These procedures followed standards set forth and approved by the University of Michigan Institutional Review Board (IRB, HUM00142680).

### Cell Culture of 3D Constructs

Membranes were soaked in 70% ethanol for 30 min, washed three times with PBS for 10 min each, and then washed with cell culture media. Cells (0.5 × 10^5^ cells/membrane) were seeded in 10 µL media. Culture media was gently added to cover the 3D constructs 1 h later.

### In Vitro Cell Migration Assay

Cells were seeded to the side of membrane constructs and cultured as described. Constructs were fixed in 4% PFA for 24 h at 4 C and embedded in paraffin for sectioning in the transverse direction to observe migration into the construct. Constructs were deparaffinized and stained with AlexaFlor‐Phalloidin and Hoesct. Sections were observed by confocal laser microscopy.

### In Vivo, Periodontal Defect

In rats, the in vivo functionality of TS‐MMS membranes to facilitate periodontal regeneration was evaluated in a periodontal defect model.^[^ ^60^, ^61^ ^]^ Male and female Fischer 344 rats, aged 10 weeks, weighing 250–290 g, were used to test membranes in vivo under an approved animal protocol (PRO00010329). Animals were anesthetized by ketamine‐xylazine and prepared with a preoperative scrub and prophylactic carprofen administration. A full‐thickness mucoperiosteal flap was elevated from a midcrestal incision to uncover alveolar bone at the mesial aspect of the maxillary first molars under a microscope. With copious irrigation, a #2 carbide round bur and slow‐speed handpiece were used to remove the alveolar bone covering the tooth's mesial root surface. After the defect was created (1.5 mm × 3 mm × 1 mm), the defect was root planed with a scaler, and a membrane was implanted into the defect location in contact with the periosteum at the margins of the defect. The flap was repositioned, and the incision was closed with cyanoacrylate; the animals recovered well and could eat a regular diet. After 4 and 8 weeks, rats were sacrificed, and the recovered bone at the defect site was evaluated by microcomputed tomography analysis.

### Microcomputed Tomography

Samples were harvested and fixed with 4% paraformaldehyde. The samples were placed in a 19 mm diameter specimen holder and scanned over their entire length using a micro‐CT system (µCT100 Scanco Medical, Bassersdorf, Switzerland) with voxel size 10 µm, 70 kVp, 114 µA, 0.5 mm AL filter, and integration time 500 ms. Vertical bone loss at each defect site was determined by measuring the distance between the alveolar bone crest and cementoenamel junction (CEJ). The CEJ was a standardized anatomical landmark for performing this 2D analysis at the broadest portion of the tooth in the buccal‐lingual dimension.^[^ ^60^, ^61^ ^]^

### Histologic Analysis

Samples prepared for the frozen section were thawed and carefully removed from OCT, which was exchanged for PBS over ten days. Maxillae samples were demineralized at 10% EDTA, 4 °C, for 4 weeks. Samples were dehydrated using an ethanol gradient and embedded in paraffin. Paraffin sections were cut at 5 µm, stained following standard histologic preparations, and imaged with a bright field microscope (Olympus). Hematoxylin and eosin (H&E) staining of paraffin sections was performed by a board‐certified histology technician (HT‐ASCP, American Society for Clinical Pathology) at the University of Michigan Oral Pathology Biopsy Service/Core Histology Laboratory according to standardized protocols in a single batch, with a control slide to ensure accurate staining. Slides were scanned to a digitized format using a Leica Aperio GT450 semi‐automated slide scanner into a virtual slide box.

### Statistical Analysis

All data were reported as mean ± standard deviation and represent a minimum sample size of n > 3. Statistical analysis was carried out in GraphPad Prism v10. Student's t‐test was used to determine the statistical significance of observed values between experimental groups where *p* < 0.05 was considered significant. All graphics note significance as: * *p* < 0.05, ** *p* < 0.01, ***p < 0.001, ****p < 0.0001.

### Ethics Statement

Procedures using primary cells and animal models followed standards set forth and approved by the University of Michigan Institutional Animal Care and Use Committee (IACUC) as approved in PRO00010329 and PRO00011263, by the ARRIVE guidelines and National Research Council's Guide for the Care and Use of Laboratory Animals.

### Declaration of Generative AI

No artificial intelligence (AI) or AI‐assisted technologies were employed in the present work.

## Conflict of Interest

The authors declare no conflict of interest.

## Author Contributions

W.S. and S.W. contributed equally to this work. W.B.S., S.M.W., M.C.B., and Y.M. performed conceptualization. W.B.S., S.M.W., R.D.F., L.D., J.A., M.E., D.N., J.X., M.C.B., and Y.M. performed data curation. W.B.S., S.M.W., R.D.F., L.D., J.X., M.C.B., and Y.M. performed formal analysis. W.B.S., M.C.B., and Y.M. performed funding acquisition. W.B.S., S.M.W., R.D.F., M.C.B., and Y.M. performed investigation. W.B.S., S.M.W., R.D.F., M.C.B., and Y.M. performed methodology. W.B.S., R.D.F., M.C.B., and Y.M. performed project administration. M.C.B. and Y.M. performed resources. W.B.S., S.M.W., R.D.F., M.C.B., and Y.M. performed software. W.B.S., M.C.B., and Y.M. performed supervision. W.B.S., S.M.W., R.D.F., M.C.B., and Y.M. performed validation. W.B.S., S.M.W., and R.D.F. performed visualization. W.B.S., S.M.W., R.D.F., M.C.B., and Y.M. wrote the original draft. W.B.S., S.M.W., R.D.F., L.D., J.A., M.E., D.N., J.X., M.C.B., and Y.M. wrote, review and edited the final manuscript.

## Supporting information



Supporting Information

## Data Availability

The data that support the findings of this study are available in the supplementary material of this article.
