# Supplementary material for: Synthetic Periodontal Guided Tissue Regeneration Membrane with Self‐Assembling Biphasic Structure and Temperature‐Sensitive Shape Maintenance
Source: Adv Healthc Mater. 2024 Oct 23;14(3):2402137. doi: 10.1002/adhm.202402137 (PMC11773119; doi:10.1002/adhm.202402137)
Supplement: Supplementary file 1 — Supporting Information [file ADHM-14-0-s001.docx]

**Supplementary Information**

**
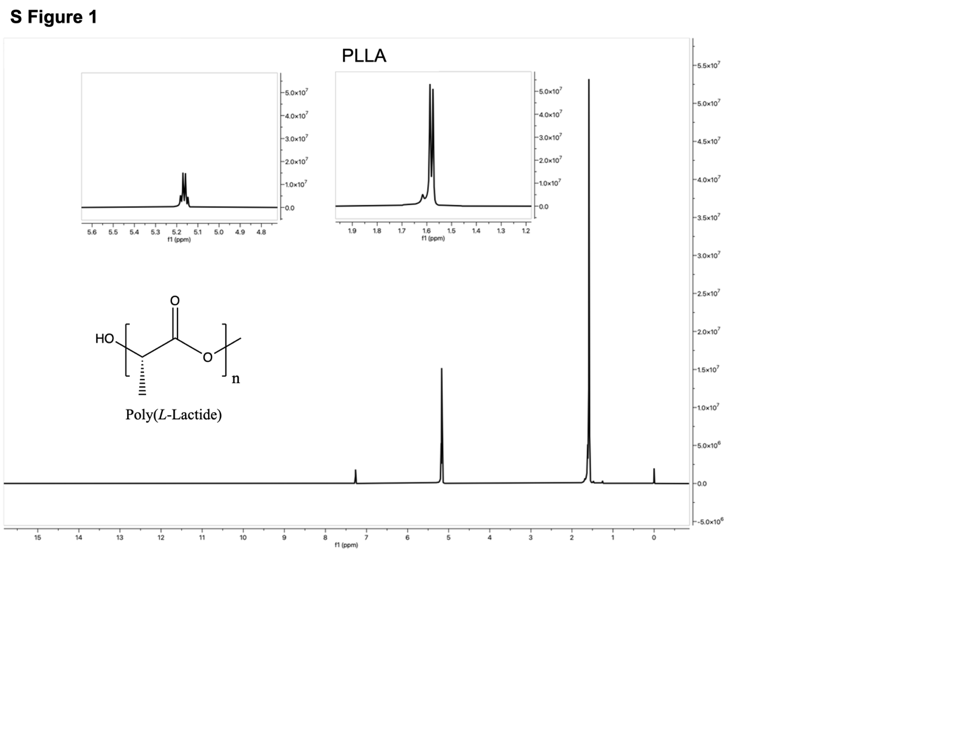
Figure S1**: ^1^H Nuclear magnetic resonance spectrum for poly (L-lactic acid), CDCl_3_.

**
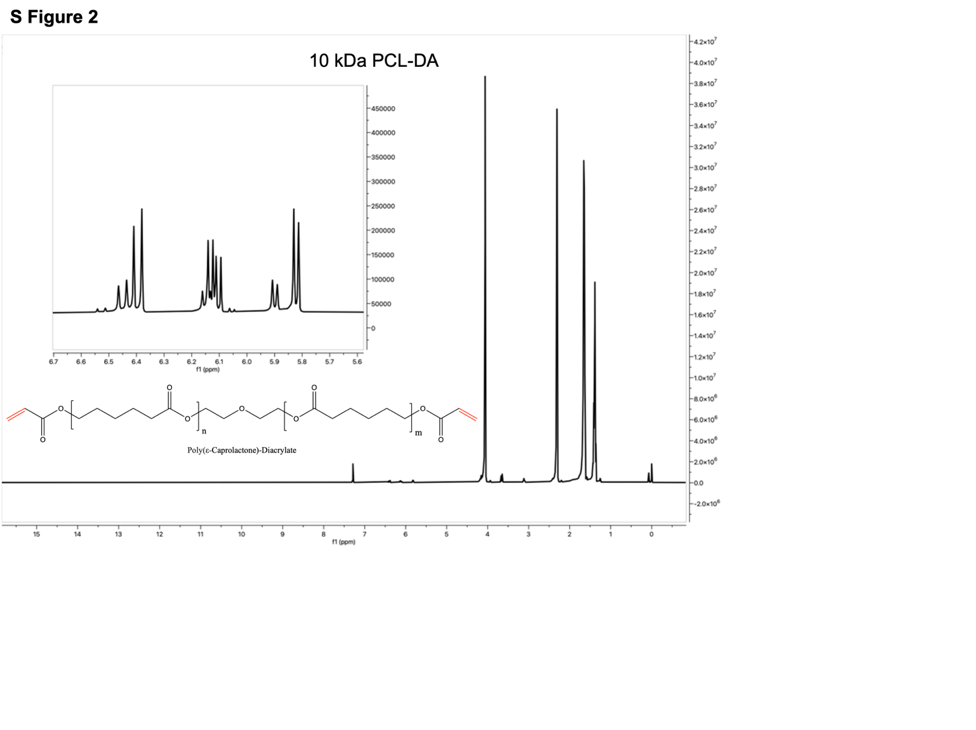
Figure S2**: ^1^H Nuclear magnetic resonance spectrum for polycaprolactone diacrylate, CDCl_3_.

**
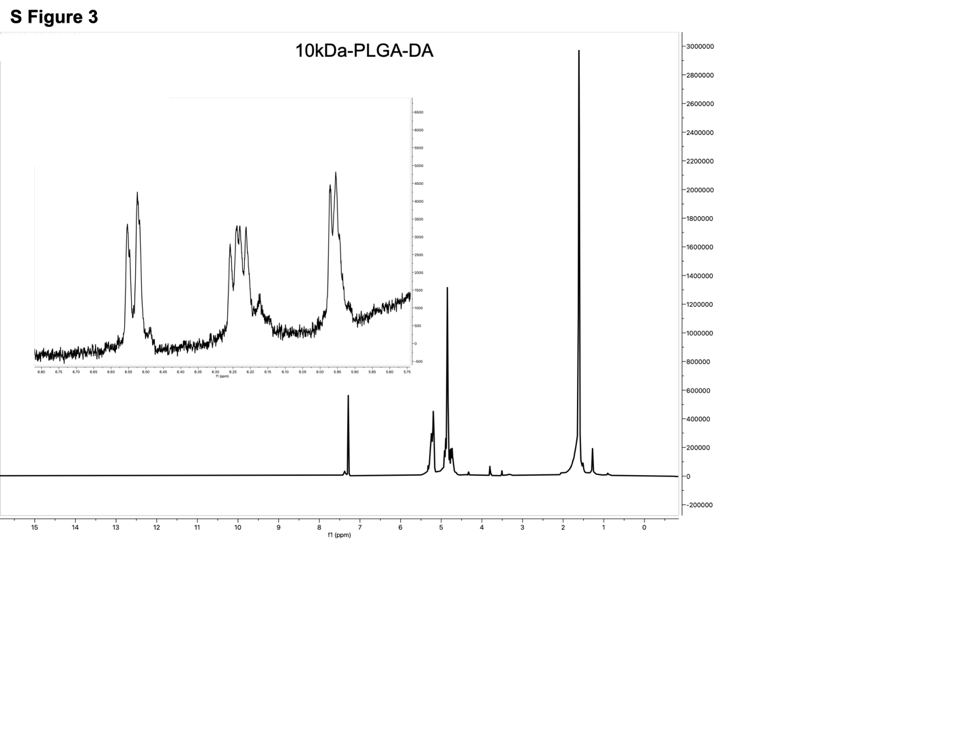
Figure S3**: ^1^H Nuclear magnetic resonance spectrum for poly (lactide-s-glycolide) diacrylate, CDCl_3_.

**
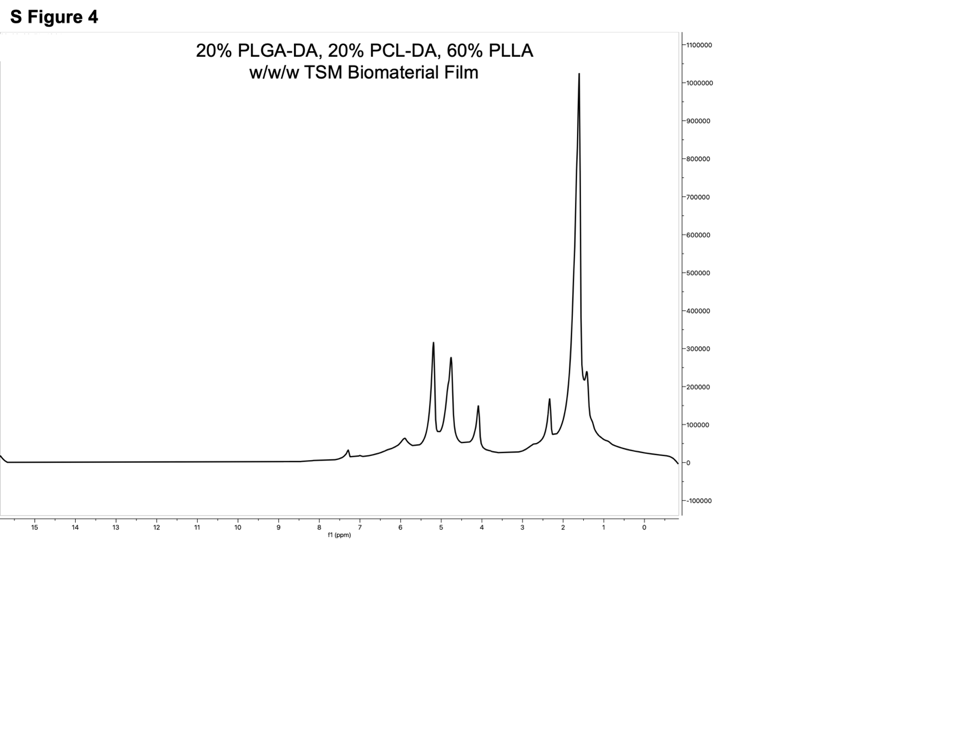
Figure S4**: ^1^H Nuclear magnetic resonance spectrum for poly (lactide-s-glycolide) diacrylate, CDCl_3_.

**
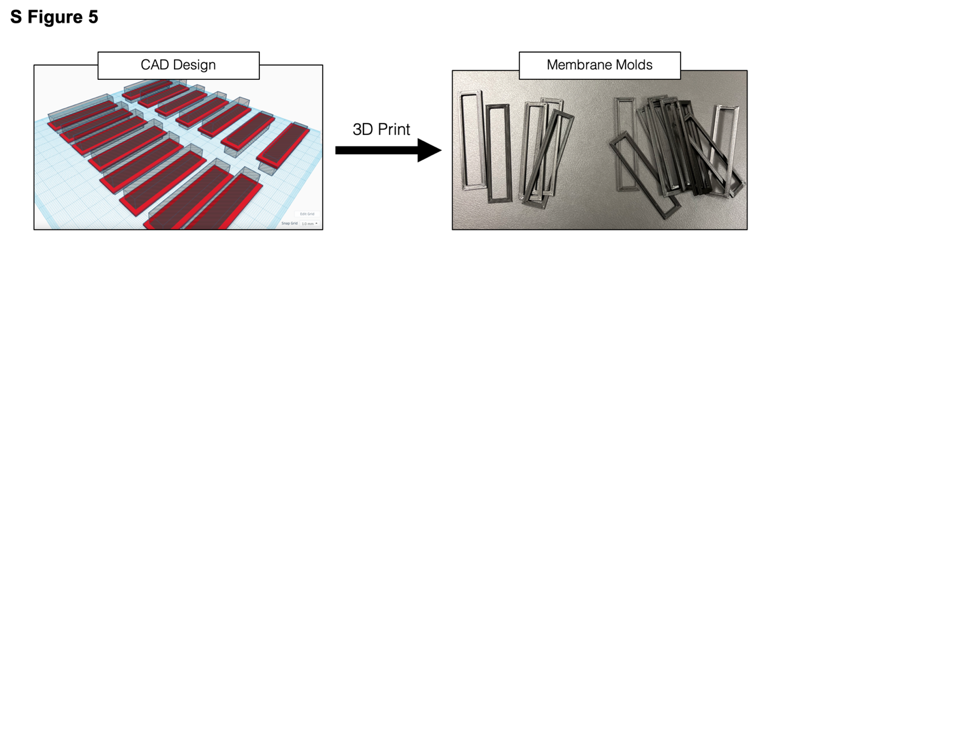
Figure S5**: Membrane molds are 3D printed from a CAD design to a specified shape and height, then glued to microscope slide glass for fabrication.

**
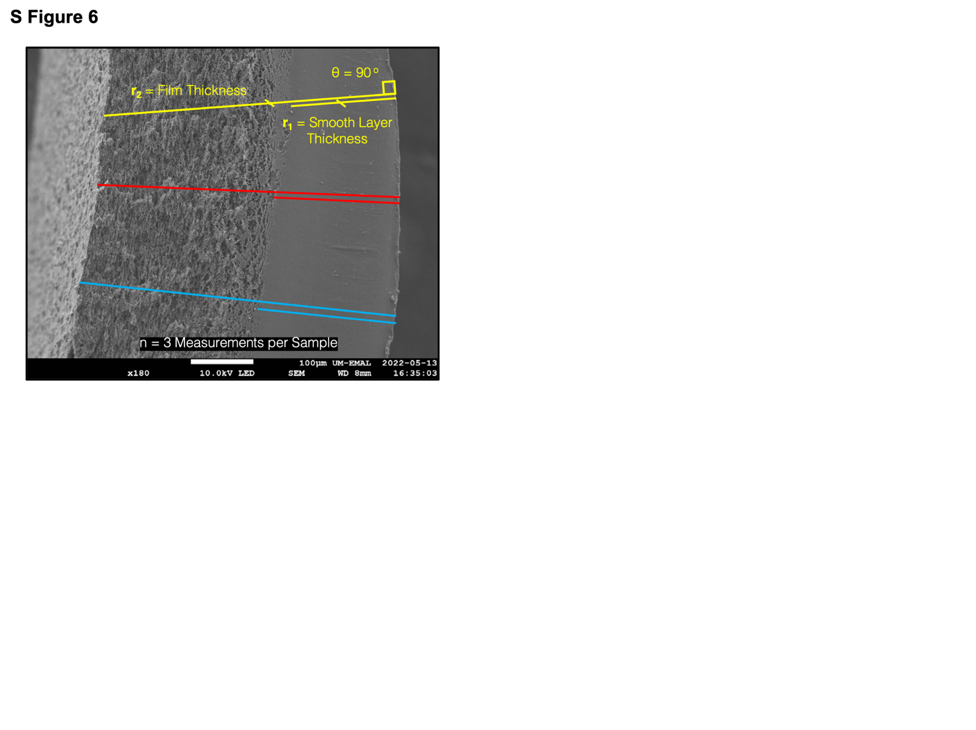
Figure S6**: Method for measuring the relative ratio of smooth (air-inhibited) to total membrane thickness from SEM images. N=3 measurements are acquired per image.

**
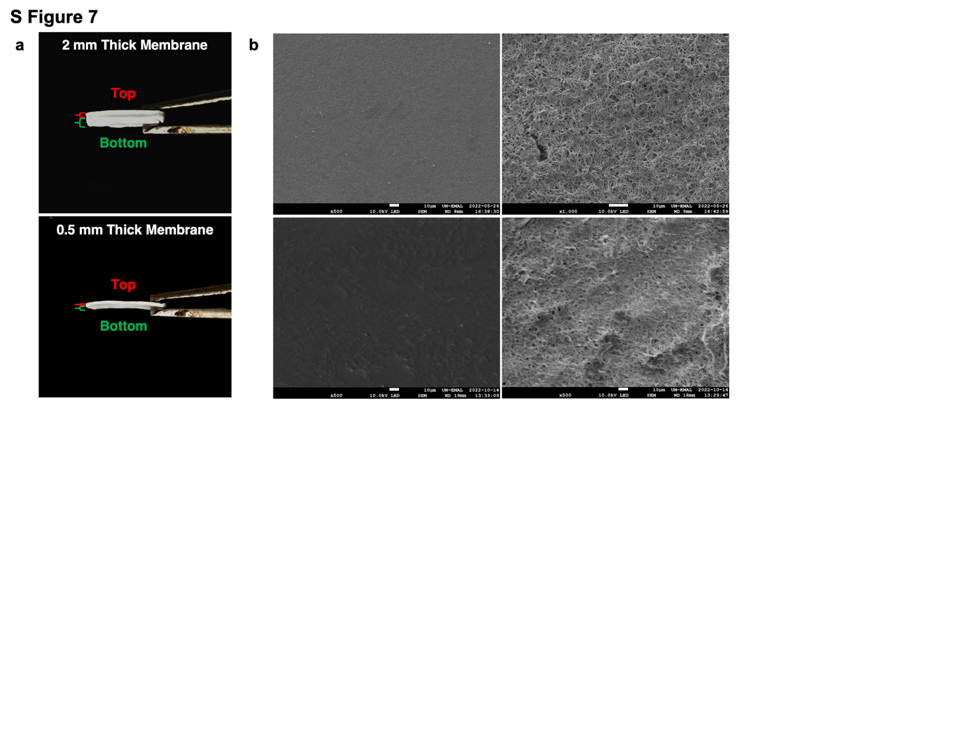
Figure S7**: The overall thickness of membranes is adjustable based on the height of the 3D-printed mold, shown at left (0.5 mm, 2 mm). As demonstrated by SEM, a bilayer is formed in each.

**
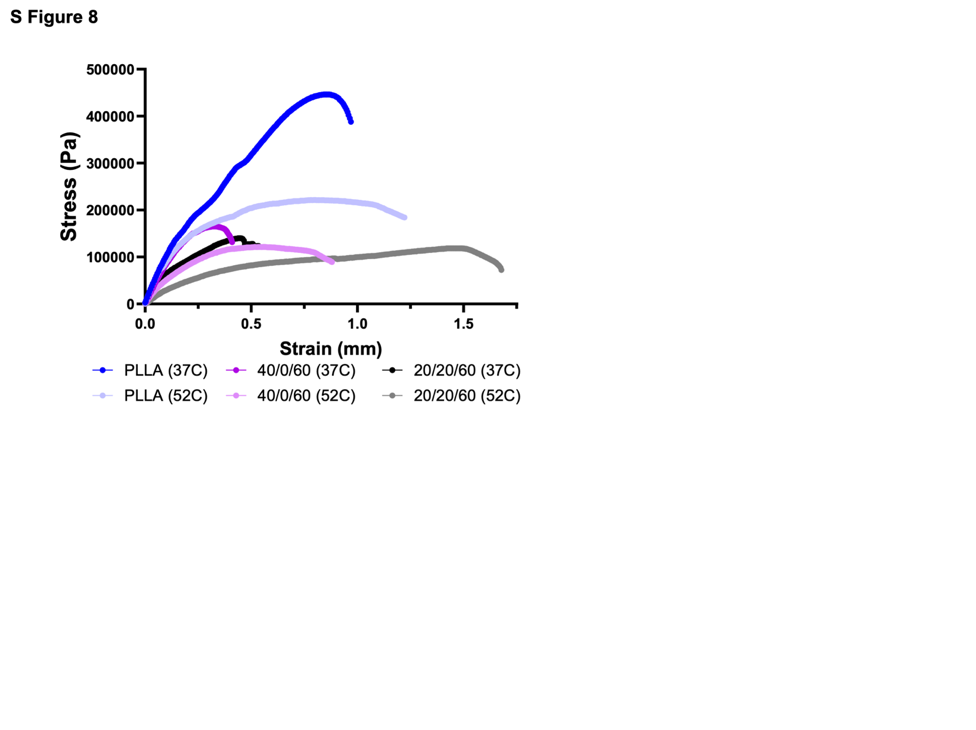
Figure S8**: Membrane mechanical properties are measured as a function of temperature and composition (D) to determine the tensile modulus (n > 5 per group)

**
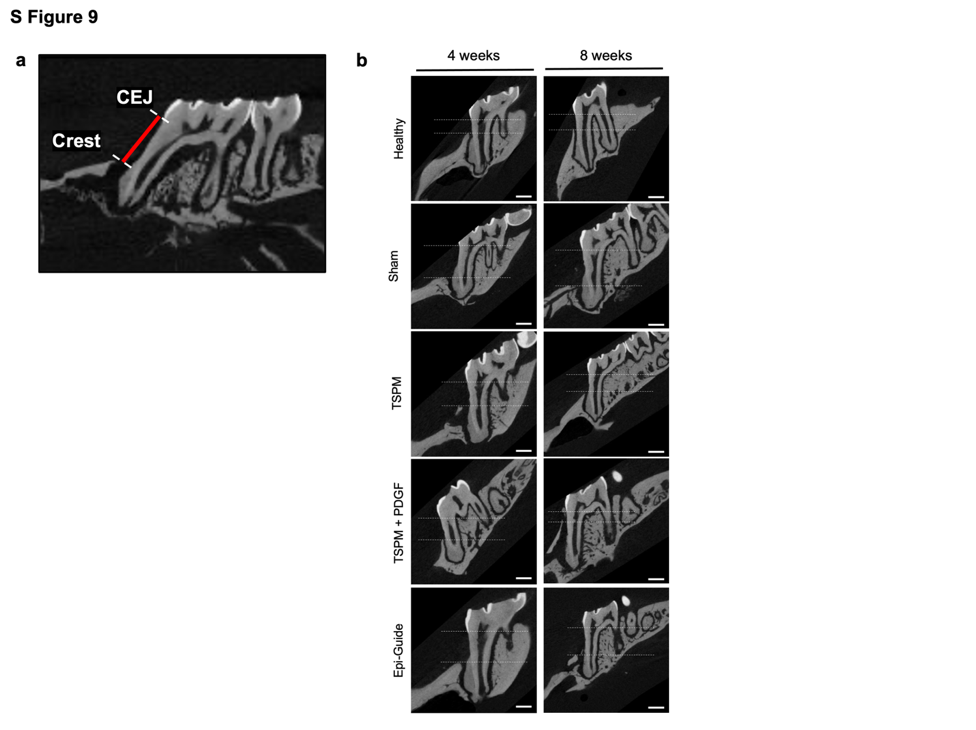
Figure S9**: Cross-sectional representative images from microcomputed tomography are used to measure the distance from the alveolar crest to the CEJ, as shown in (a), at the widest portion of the maxillary first molar in the buccal-lingual direction. Representative images at each time point are shown in (b). Scale: 1 mm.

**
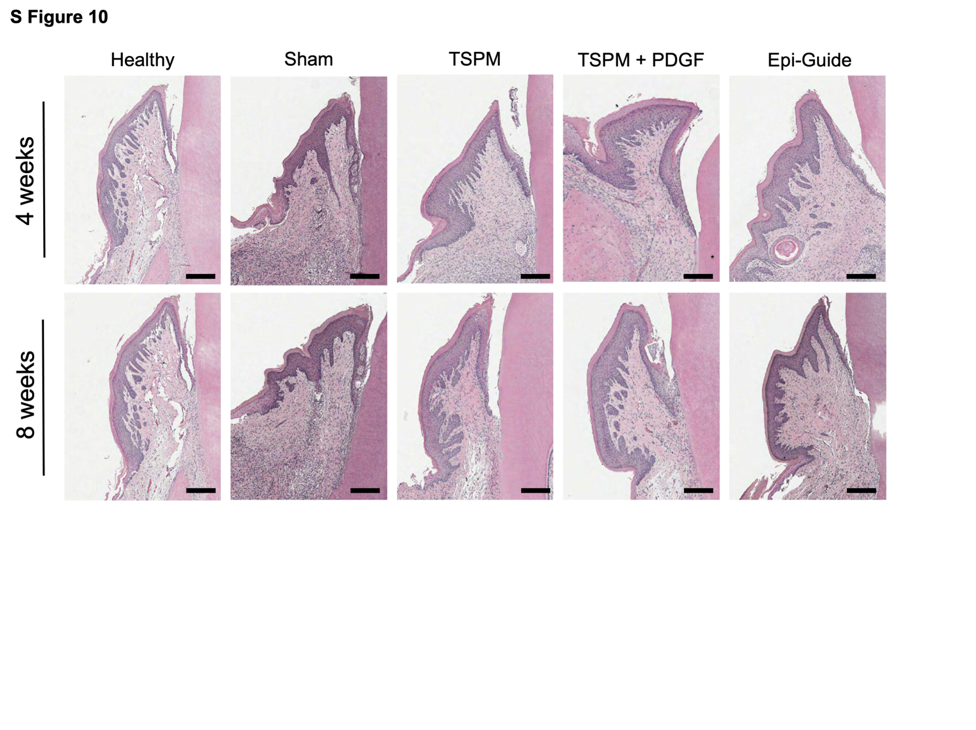
Figure S10**: Hematoxylin and eosin staining of the masticatory mucosa overlying the defect site is shown at 4 weeks (top) and 8 weeks (bottom). Scale: 250 um.
